# Study of Sexual Dimorphism in Metatarsal Bones: Geometric and Inertial Analysis of the Three-Dimensional Reconstructed Models

**DOI:** 10.3389/fendo.2021.734362

**Published:** 2021-10-14

**Authors:** Yaming Liu, Djorde Antonijević, Ruining Li, Yuxuan Fan, Ksenija Dukić, Milutin Mićić, Genyu Yu, Zhiyu Li, Marija Djurić, Yifang Fan

**Affiliations:** ^1^ Foot Research Laboratory, School of Physical Education and Sport Science, Fujian Normal University, Fuzhou, China; ^2^ Laboratory for Anthropology, Institute for Anatomy, School of Medicine, University of Belgrade, Belgrade, Serbia; ^3^ Laboratory for Atomic Physics, Institute for Nuclear Science “Vinca”, University of Belgrade, Belgrade, Serbia; ^4^ School of Dental Medicine, University of Belgrade, Belgrade, Serbia; ^5^ College of Foreign Studies, Jinan University, Guangzhou, China

**Keywords:** metatarsal, cross section, 3D reconstruction, principal moments of inertia, sex determination

## Abstract

The aim of the present paper is to determine the sex of the individual using three-dimensional geometric and inertial analyses of metatarsal bones. Metatarsals of 60 adult Chinese subjects of both sexes were scanned using Aquilion One 320 Slice CT Scanner. The three-dimensional models of the metatarsals were reconstructed, and thereafter, a novel software using the center of mass set as the origin and the three principal axes of inertia was employed for model alignment. Eight geometric and inertial variables were assessed: the bone length, bone width, bone height, surface-area-to-volume ratio, bone density, and principal moments of inertia around the *x*, *y*, and *z* axes. Furthermore, the discriminant functions were established using stepwise discriminant function analysis. A cross-validation procedure was performed to evaluate the discriminant accuracy of functions. The results indicated that inertial variables exhibit significant sexual dimorphism, especially principal moments of inertia around the *z* axis. The highest dimorphic values were found in the surface-area-to-volume ratio, principal moments of inertia around the *z* axis, and bone height. The accuracy rate of the discriminant functions for sex determination ranged from 88.3% to 98.3% (88.3%–98.3% cross-validated). The highest accuracy of function was established based on the third metatarsal bone. This study showed for the first time that the principal moment of inertia of the human bone may be successfully implemented for sex estimation. In conclusion, the sex of the individual can be accurately estimated using a combination of geometric and inertial variables of the metatarsal bones. The accuracy should be further confirmed in a larger sample size and be tested or independently developed for distinct population/age groups before the functions are widely applied in unidentified skeletons in forensic and bioarcheological contexts.

## 1 Introduction

“Virtopsy” is a term introduced in forensics and bio-archeology to describe the application of the three-dimensional (3D) cross-section imaging (CSI) analysis of human remains. These techniques include organs’ 3D reconstruction and precise quantitative measurements based on multi-slice computed tomography or magnetic resonance imaging data (1). Indeed, the replacement of traditional postmortem techniques with CSI examination has been recommended by scientific, cultural, and humanitarian groups due to its non-invasiveness, digital nature, and 3D reconstruction opportunities (2). Literature data provide numerous evidences that CSI reconstruction is useful for the analysis of the neuronal morphology, bones, and teeth (3–8). For instance, the possibility to estimate a person’s age by assessment of dental pulp volume or sex by calculating long bone’s metrical quantities has been documented (9–13).

Sex estimation of skeletal remains is a basic element in creating a biological profile in archeology and forensics (14, 15). The estimation relies heavily on the analysis of the pelvic and cranial features (7, 16). However, in reality, it is not rare that the discovered skeletal remains are incomplete and consequently investigators have to focus on the remaining bones (17). In this context, metatarsal bones with small quantity and surface area are more likely to be preserved intact and in some cases they present the only option for sex estimation (18). The prevalence of metatarsal bones at archeological sites ranges between 43% and 89%, considerably more than that of other bones. For example, in seven forensics cases in Northern Italy 97.1% metatarsal bones were present, including 100% first metatarsal bones (19).

Research has already proven the accuracy of virtual sex assessment using CSI of metatarsal bones. For instance, metatarsals’ linear measurements such as maximum length, width of head, and width of base are shown to provide accurate sex estimation (17). In addition, the volume of the first metatarsal bone and the torsion of the second metatarsal bone were employed to establish successful sex and aging protocols (19, 20). The accuracy of currently available geometric protocols is approximately 80%, which gives room for further improvements (19). Sex estimation can be accurately performed using the DNA analysis, which highly depends on the quantity and quality of DNA samples; however, it is not an applicable method to accurately identify sex of human skeletal remains, since the DNA begins to degrade immediately after the cells die (21). Although bone and teeth can provide some protection against DNA degradation, the environment for preservation is highly demanding, such as temperature, moisture levels, oxygen levels, soil composition, and pH value (22). Y chromosome deletions or mutations in the priming or binding sites can lead to incorrect estimation of sex and reduce the accuracy of DNA analysis (23–25). Studies show that the accuracy of DNA analysis in determining the sex of ancient human remains ranges from 52% to 95% (23–25). For bone fossils, the older the fossil, the lower the amount of extractable DNA. No extractable DNA was left in bone fossil between 200,000 and 500,000 years ago (26). Furthermore, the process of DNA extraction is destructive (22, 27), which is not feasible when preservation of ancient skeletal remains is required (28). Therefore, when we attempt to determine the sex of bone fossils, the advantages of morphology and inertia variables will be more obvious.

The current investigation seeks to test the hypothesis that 3D reconstruction of metatarsal bones might present a promising alternative to traditionally employed methods for forensics and archeological sex estimation. More precisely, this study aims to define physical metatarsal bone quantities of interest for the discrimination between male and female subjects. To this purpose, 60 subjects’ metatarsals (n = 600) were scanned using computed tomography and virtually analyzed with an intention to identify quantitative measurements referring to the sex of the individual. In addition to normalizing bone’s sexual identifying geometric variables (bone length, width, height, surface area, and volume), this study was specifically designed to increase the sex determination accuracy by including the inertial variables—three principal moments of inertia (PMI) relative to their principal axes of inertia (PAI) (*x*, *y*, and *z*).

## 2 Subjects and Methods

### 2.1 Subjects

We recruited subjects from our university who volunteered to participate in this program. Sixty healthy adults (30 males and 30 females) from Fujian Normal University were selected. Their mean age was 20.9 ± 3.0 years, mean height 170.9 ± 9.9 cm, and mean weight 62.5 ± 10.6 kg. The detailed characteristics of male and female subjects are shown in **Table S1** (**Supplementary File III**). The study received approval from the Ethical Committee of Fujian Normal University. The subjects provided fully informed consent to participate in the study by signing a written consent form. Then, a questionnaire was distributed to volunteer students to exclude those with lower limb injury history. Each potential subject’s annual medical report was checked to exclude those with disease or trauma in their nervous and/or musculoskeletal system.

### 2.2 Scanning Procedure

Subjects were scanned using Aquilion One 320 Slice CT Scanner (Toshiba, Japan). The scan settings were as follows: tube voltage of 120 kV, tube current exposure time of 50 mAs, layer distance of 0.45–50 mm, pixel size of 0.46 ± 0.02 mm, and automatic threshold between -1024 and 4145 Hounsfield units (HU). The scanning was conducted along the transect of both feet, from top to bottom. The scanning posture of 60 subjects is shown in **Supplementary File I**; **Figures S1A, B**.

### 2.3 Definition of Coordinate System of Metatarsal Bones

The 3D models were constructed using *Mimics* software system (Mimics Research 17.0 for X64; Materialise, Leuven, Belgium). The reconstructed metatarsal bones are shown in **Supplementary File I**; **Figures S2A, B**. Software solution was employed to position the 3D models of 600 metatarsals (**Supplementary File II**). In brief, software includes setting the direction and order of three coordinate axes of the metatarsal. Specifically, by going through the center of mass (COM) of the metatarsal, the PAI set to go from metatarsal head to base was the *z* axis, with the direction from the head to the base as the positive direction; the PAI set to go from plantar to dorsal was the *x* axis; and the PAI set to go from the medial metatarsal body to lateral was the *y* axis.

The bone length, width, height, bone density, surface area, volume, and three PMIs around the *x*, *y*, and *z* axes were obtained from the positioned metatarsal bones (**Supplementary File I**; **Figures S3A, B**).

### 2.4 Extracting Biometric Sex Estimation Identifiers

The bone length, width, height, surface area, and volume were extracted automatically from *Mimics* software (**Supplementary File I**; **Figure S4**) and from the 3D models of metatarsal bones described in Section 2.3.

#### 2.4.1 Normalization of Linear Variables

Equation (1) was used to normalize the linear measurements and to eliminate the effect from subjects’ body height difference, possible sub-voxel scanning accuracy (29, 30), and possible voxel order of magnitude errors from segmentation accuracy derived from both segmentations alone or non-detected subject micro-movement during the scanning procedure (31):


(1) $$\{\begin{array}{l} L_{n}=\frac{L_{p}}{L_{p}+W_{p}+H_{p}}\times 100\\ W_{n}=\frac{W_{p}}{L_{p}+W_{p}+H_{p}}\times 100\\ H_{n}=\frac{H_{p}}{L_{p}+W_{p}+H_{p}}\times 100 \end{array}$$

where *Lp*, *Wp*, and *Hp* refer to the length, width, and height of the positioned bone, and *Ln*, *Wn*, and *Hn* those of the normalized, respectively.

#### 2.4.2 Normalization of Inertial Variables

The Hounsfield number of a CT scan is a product of radiation dose and attenuation coefficient (derived from density and atomic number) of the scanned material (32). In our case, it is influenced by bone density, body mass and size, and again possible micro-movement of the subjects during scanning procedure, which can create voxel order of magnitude geometric dimension errors on the 3D model derived from the scan by altering the valve of HU and thus threshold and segmentation procedures. As we derive the mass of 3D models of metatarsal bones from the HU used for the segmentation procedure to calculate the PAI, Equation (2) was employed to eliminate those effects (31) and to normalize inertial variables:


(2) $$\{\begin{array}{l} PMI_{x}=\frac{PMI_{x}}{PMI_{x}+PMI_{y}+PMI_{z}}\times 100\\ PMI_{y}=\frac{PMI_{y}}{PMI_{x}+PMI_{y}+PMI_{z}}\times 100\\ PMI_{z}=\frac{PMI_{z}}{PMI_{x}+PMI_{y}+PMI_{z}}\times 100 \end{array}$$

#### 2.4.3 Calculation of Surface-Area-to-Volume Ratio

The surface-area-to-volume ratio (SA: V) of 3D reconstruction of the metatarsal bone was calculated, as shown in Equation (3):


(3) $$SA:V=\frac{S}{V}$$

where *S* refers to the surface area of metatarsal bone, and *V* to the volume of metatarsal bone.

#### 2.4.4 Calculation of Bone Density

The bone density of the 3D reconstructed metatarsal bone was calculated, shown in Equation (4):


(4) $$d=\frac{\int_{1}^{N}d_{i}}{N}$$

where $d_{i}=\frac{g_{i}}{g_{w}}$ , *g_i_* stands for the gray value of the volume element, *g_w_* that of water. The equipment has been calibrated; the gray value of the air is set to 0, and that of the water is 1024. N refers to the number of bone’s volume elements.

### 2.5 Parameter Setting of the Reconstruction

The same parameter settings were used to reconstruct all metatarsal bones. Specifically, in the *Mimics* software, the “Predefined Thresholds Sets: Bone (CT),” “Fill holes,” and “Keep largest” options of “Thresholding” were not selected. The operations of “Local Thresholding,” “Region Growing,” and “Dynamic Region Growing” were not performed. In “Morphology Operations,” the operation was set to “Close” to operate the selected metatarsal.

### 2.6 Statistical Analysis

To test the influence of the reconstruction parameter setting on the consistency of reconstructed metatarsal geometric measurements, intraclass correlation coefficient (ICC) analysis was performed on the length, width, height, surface area, volume, and SA: V of 60 metatarsal bones from previous research (33), where the reconstruction parameter settings were the same as this study, and 60 metatarsal bones were scanned and reconstructed twice.

The assumption of normality and homogeneity of variances were tested by the Shapiro–Wilk test and Levene’s test, respectively (**Supplementary File III**; **Tables S3**–**S5**). The comparisons of measurement values between sexes were evaluated with the independent sample *t*-test analysis when data were normal distribution and homoscedasticity. The Mann–Whitney U test was performed when data were non-normal distribution, and the results of Welch’s test would be accepted when data were heteroscedasticity. The statistical level was determined as *p* < 0.05. The sexual dimorphism index (SDI) was determined as (X*m* - X*f/*X*m* + X*f*) × 100, where X*m* and X*f* are the mean values of the male and female groups, respectively (34). SDI represents the degree of variation between sexes. When males’ variables were larger than those of the females’, the SDI value was positive; otherwise, it was negative. The closer it got to zero, the less significant the difference between the male and the female was. The correlation between subjects’ characteristics and bone variables was evaluated by Spearman’s correlation coefficient (35).

To determine the best sex-discriminatory variables, the stepwise discriminant function analysis (SDFA) (Wilk’s lambda) was performed for each left and right metatarsal bone. The assumption tests including multivariate normality, multicollinearity, multivariate outliers within groups, homogeneity of variances/covariances, and linearity were conducted prior to the performance of the SDFA. Multivariate normality was assessed by Mardia’s skewness and kurtosis (36). A Mahalanobis distance test was used to detect multivariate outliers (37). The Pearson correlation test was performed to test multicollinearity among variables (38). Homogeneity of variance–covariance matrices and linearity were evaluated by Box’s M test and matrix scatter plots, respectively. Data analyses were processed with *SPSS 23.0* (IBM Corp.).

Prior probability was set as “all group equal” for all analyses. The smaller value of the function’s Wilks’ lambda indicates greater discriminatory ability of the function. The standardized canonical discriminant coefficients imply contribution of each variable. The higher the value, the greater the contribution of the variable. The structure matrix demonstrates the correlation between each variable and the discriminant function. The closer the value of the variable to 1, the stronger the correlation. Unstandardized coefficients are utilized to form the discriminant function and calculate the discriminant function score (Y). The form of function is Y = *a*_1_ *x*_1_ + *a*_2_ *x*_2_ +···+ *a_n_x_n_* + *C* (a_1_ - a_n_ = unstandardized coefficients, x_1_-x_n_ = variables, n = the number of variables, and C = the constant value). A “leave one out classification” procedure is performed in order to estimate the accuracy rate of the original sample and the sample created by cross-validation.

## 3 Results

**Figure 1:**
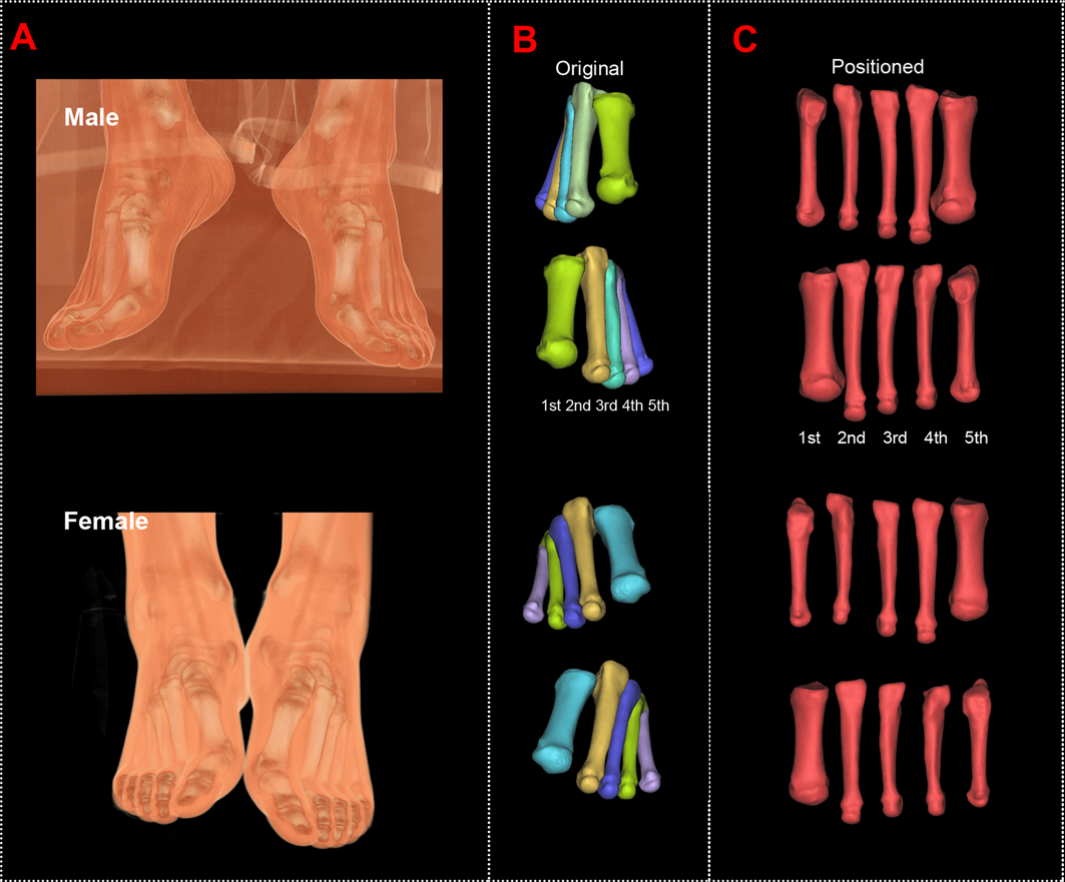
Computed tomography reconstructions of the original and positioned scanning posture of the investigated metatarsal bones. **(A)** Scanning postures of a male and a female subject. **(B)** Reconstructed first to fifth metatarsal bones of the male and female subjects. **(C)** Positioned first to fifth metatarsal bones of the male and female subjects.

**Figure 2:**
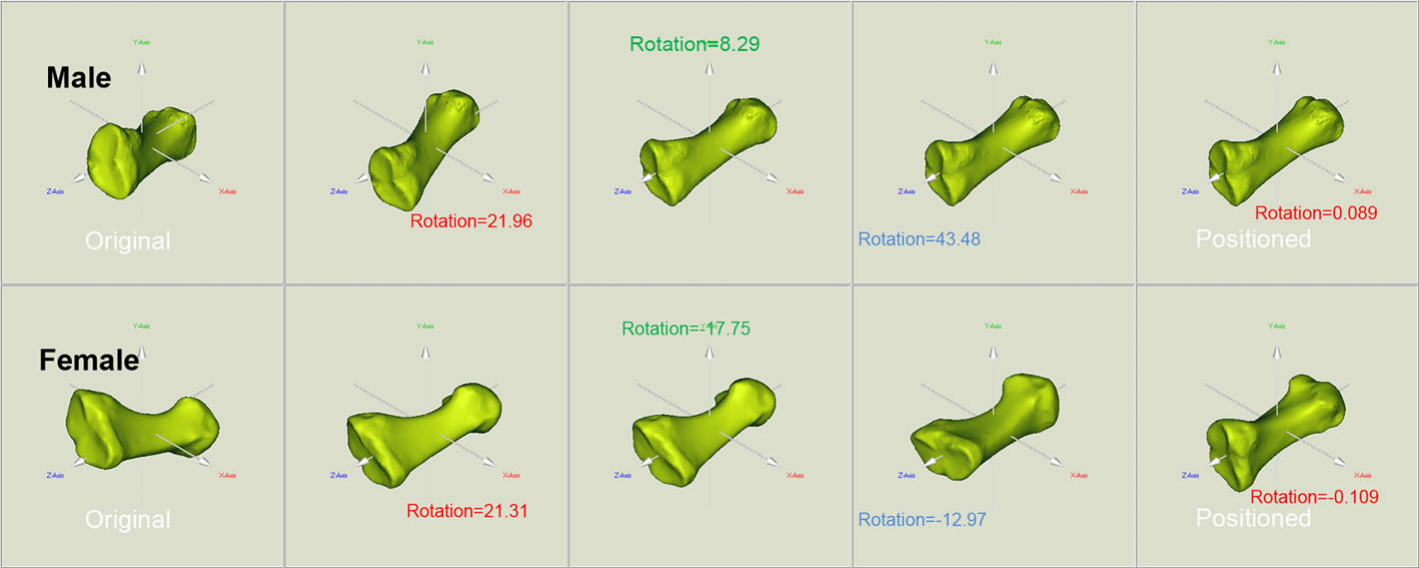
Positioning of the reconstructed bone alongside the body coordinate system of the male and female metatarsals. To facilitate the location and orientation positioning, COMs were aligned and the PAIs were used to define the coordinate system within each metatarsal. The rotation around the *x*, *y*, and *z* axes allows to achieve the alignment of the investigated metatarsals.

The original and positioned scanning postures of the investigated metatarsal bones are shown in **Figure 1**. **Figure 2** shows the process of bones’ alignment in the *x*, *y*, and *z* planes.

Shown in **Table S2** are the ICC analysis results of the length, width, height, surface area, volume, and SA: V of the reconstructed metatarsal bones from two scans. The best consistency is shown in bone length, up to 1.00. The volume of the third metatarsal presents the lowest ICC, i.e., 0.81. The ICC values of the remaining measurements range from 0.91 to 0.99. It is worth noting that the ICC values of SA: V are between 0.93 and 0.98, higher than those of surface area and volume.

**Tables 1**–**3** reveal the descriptive results and SDI values of eight variables of both sides of the investigated metatarsal bones between sexes. The highest SDIs were found in SA: V (-6.055% – -7.656% and -6.227 – - 7.949% from the left and right sides, respectively), PMI*z* (3.797%–5.455% and 2.564%–7.692% from the left and right sides, respectively), and height (1.292%–2.389% and 1.026%–2.235% from the left and right sides, respectively). SA: V and PMI*z* show greater sexual dimorphism than linear variables. Of note is that the SDI values of bone length, SA: V, PMI*x*, and PMI*y* were negative, indicating a larger value of females than that of males.

**Table 1:** Descriptive results of normalized length, width, and height of metatarsal bone *in vivo* based on its PAI.

Metatarsals	Geometric variables		Male					SDI (%)	Female				
			Mean	SD	Min	Max	95% CI		Mean	SD	Min	Max	95% CI
**1st**	**Length**	**L**	0.553	0.014	0.528	0.577	0.548–0.558	-0.896	0.563	0.011	0.543	0.586	0.559–0.567
		**R**	0.550	0.013	0.525	0.574	0.545–0.555	-1.168	0.563	0.011	0.542	0.583	0.559–0.567
	**Width**	**L**	0.251	0.008	0.225	0.265	0.247–0.254	0.803	0.247	0.006	0.238	0.265	0.244–0.249
		**R**	0.253	0.008	0.224	0.269	0.250–0.256	1.403	0.246	0.005	0.233	0.255	0.244–0.248
	**Height**	**L**	0.196	0.008	0.182	0.214	0.193–0.199	1.554	0.190	0.009	0.174	0.210	0.187–0.193
		**R**	0.197	0.008	0.182	0.215	0.194–0.200	1.546	0.191	0.008	0.174	0.202	0.187–0.194
**2nd**	**Length**	**L**	0.659	0.015	0.629	0.687	0.653–0.664	-0.528	0.666	0.010	0.639	0.686	0.663–0.670
		**R**	0.658	0.015	0.624	0.693	0.652–0.663	-0.529	0.665	0.011	0.637	0.681	0.661–0.669
	**Width**	**L**	0.145	0.008	0.133	0.164	0.142–0.148	0.694	0.143	0.006	0.132	0.157	0.141–0.145
		**R**	0.145	0.008	0.129	0.162	0.142–0.148	1.045	0.142	0.007	0.122	0.157	0.140–0.145
	**Height**	**L**	0.196	0.010	0.178	0.220	0.192–0.200	1.292	0.191	0.008	0.178	0.218	0.188–0.194
		**R**	0.197	0.010	0.173	0.219	0.193–0.201	1.026	0.193	0.008	0.176	0.207	0.190–0.195
**3rd**	**Length**	**L**	0.663	0.013	0.636	0.687	0.658–0.668	-0.674	0.672	0.010	0.650	0.690	0.668–0.676
		**R**	0.663	0.014	0.634	0.684	0.658–0.668	-0.749	0.673	0.012	0.647	0.692	0.668–0.677
	**Width**	**L**	0.139	0.008	0.125	0.156	0.137–0.142	1.091	0.136	0.006	0.123	0.152	0.134–0.138
		**R**	0.139	0.008	0.128	0.156	0.137–0.142	1.091	0.136	0.007	0.121	0.152	0.133–0.138
	**Height**	**L**	0.198	0.009	0.178	0.222	0.195–0.201	1.538	0.192	0.008	0.173	0.206	0.189–0.195
		**R**	0.198	0.009	0.180	0.221	0.194–0.201	1.538	0.192	0.009	0.172	0.213	0.188–0.195
**4th**	**Length**	**L**	0.670	0.012	0.645	0.696	0.666–0.674	-0.741	0.680	0.010	0.659	0.699	0.677–0.684
		**R**	0.668	0.013	0.638	0.697	0.664–0.673	-0.890	0.680	0.011	0.656	0.697	0.676–0.684
	**Width**	**L**	0.148	0.007	0.135	0.166	0.146–0.151	0.680	0.146	0.008	0.135	0.168	0.143–0.149
		**R**	0.149	0.006	0.138	0.159	0.147–0.151	1.361	0.145	0.007	0.127	0.160	0.142–0.148
	**Height**	**L**	0.182	0.010	0.160	0.209	0.178–0.185	2.247	0.174	0.008	0.161	0.189	0.171–0.177
		**R**	0.183	0.010	0.163	0.213	0.179–0.186	2.235	0.175	0.007	0.161	0.192	0.172–0.178
**5th**	**Length**	**L**	0.658	0.014	0.630	0.706	0.653–0.663	-0.679	0.667	0.012	0.642	0.692	0.663–0.672
		**R**	0.661	0.013	0.637	0.701	0.656–0.665	-0.527	0.668	0.010	0.642	0.693	0.664–0.672
	**Width**	**L**	0.192	0.010	0.163	0.207	0.188–0.196	0.524	0.190	0.008	0.173	0.205	0.187–0.192
		**R**	0.191	0.011	0.164	0.206	0.187–0.195	0.526	0.189	0.007	0.175	0.203	0.187–0.192
	**Height**	**L**	0.150	0.008	0.131	0.173	0.147–0.153	2.389	0.143	0.007	0.130	0.158	0.141–0.146
		**R**	0.148	0.008	0.134	0.168	0.145–0.151	1.718	0.143	0.007	0.130	0.155	0.140–0.145

PAI, principal axes of inertia; SDI, sexual dimorphism index; 95% CI, 95% confidence interval of difference.

All linear variables were normalized by Equation (1).

**Table 2:** Descriptive results of SA: V (mm^-1^) and bone density (HU/1024) of metatarsal bone *in vivo*.

Metatarsals	Geometric variables		Male					SDI (%)	Female				
			Mean	SD	Min	Max	95% CI		Mean	SD	Min	Max	95% CI
**1st**	**SA: V**	**L**	0.256	0.016	0.224	0.297	0.250–0.262	-6.055	0.289	0.014	0.262	0.315	0.284–0.294
		**R**	0.256	0.016	0.223	0.299	0.250–0.262	-6.227	0.290	0.013	0.263	0.315	0.285–0.295
	**Density**	**L**	1.602	0.059	1.452	1.718	1.580–1.624	0.786	1.577	0.054	1.436	1.670	1.557–1.597
		**R**	1.605	0.072	1.462	1.744	1.578–1.632	0.690	1.583	0.056	1.443	1.681	1.462–1.744
**2nd**	**SA: V**	**L**	0.362	0.022	0.328	0.420	0.354–0.371	-7.417	0.420	0.026	0.359	0.470	0.411–0.430
		**R**	0.359	0.020	0.332	0.413	0.351–0.367	-7.949	0.421	0.027	0.366	0.487	0.411–0.431
	**Density**	**L**	1.710	0.078	1.522	1.907	1.681–1.739	-1.099	1.748	0.077	1.592	1.909	1.720–1.777
		**R**	1.710	0.085	1.522	1.913	1.678–1.742	-0.581	1.730	0.069	1.586	1.860	1.705–1.756
**3rd**	**SA: V**	**L**	0.386	0.018	0.348	0.417	0.379–0.393	-7.656	0.450	0.021	0.407	0.502	0.442–0.457
		**R**	0.383	0.016	0.346	0.407	0.377–0.389	-7.822	0.448	0.022	0.409	0.504	0.440–0.456
	**Density**	**L**	1.661	0.077	1.502	1.844	1.633–1.690	-0.150	1.666	0.068	1.514	1.777	1.641–1.692
		**R**	1.660	0.075	1.496	1.803	1.632–1688	0.242	1.652	0.064	1.516	1.775	1.628–1.676
**4th**	**SA: V**	**L**	0.377	0.016	0.341	0.418	0.371–0.383	-7.371	0.437	0.023	0.392	0.487	0.428–0.445
		**R**	0.375	0.016	0.335	0.408	0.368–0.381	-7.635	0.437	0.023	0.392	0.484	0.428–0.446
	**Density**	**L**	1.631	0.077	1.429	1.760	1.602–1.660	0.215	1.624	0.061	1.458	1.751	1.601–1.647
		**R**	1.633	0.071	1.429	1.731	1.607–1.660	0.400	1.620	0.053	1.482	1.718	1.600–1.639
**5th**	**SA: V**	**L**	0.346	0.017	0.313	0.388	0.340–0.353	-6.989	0.398	0.023	0.354	0.444	0.389–0.406
		**R**	0.345	0.016	0.307	0.370	0.339–0.351	-7.133	0.398	0.023	0.358	0.450	0.389–0.406
	**Density**	**L**	1.682	0.077	1.466	1.798	1.654–1.711	0.478	1.666	0.053	1.569	1.813	1.647–1.686
		**R**	1.682	0.070	1.466	1.788	1.655–1.708	0.870	1.653	0.053	1.549	1.800	1.633–1.672

SDI, sexual dimorphism index; 95% CI, 95% confidence interval of difference.

SA: V was calculated by Equation (3). The bone density was calculated by Equation (4).

**Table 3:** Descriptive results of three normalized PMIs of metatarsal bone *in vivo* based on its PAI.

Metatarsals	Inertial variables		Male					SDI (%)	Female				
			Mean	SD	Min	Max	95% CI		Mean	SD	Min	Max	95% CI
**1st**	**PMI*x***	**L**	0.462	0.005	0.446	0.470	0.459–0.464	-0.431	0.466	0.003	0.460	0.471	0.464–0.467
		**R**	0.461	0.006	0.447	0.470	0.459–0.463	-0.432	0.465	0.003	0.460	0.471	0.464–0.467
	**PMI*y***	**L**	0.452	0.005	0.444	0.467	0.450–0.454	-0.550	0.457	0.004	0.449	0.467	0.455–0.458
		**R**	0.451	0.005	0.443	0.463	0.449–0.453	-0.551	0.456	0.003	0.450	0.466	0.455–0.458
	**PMI*z***	**L**	0.087	0.009	0.064	0.099	0.084–0.090	5.455	0.078	0.006	0.063	0.089	0.075–0.080
		**R**	0.088	0.009	0.067	0.103	0.085–0.091	6.024	0.078	0.005	0.068	0.089	0.076–0.080
**2nd**	**PMI*x***	**L**	0.478	0.003	0.472	0.485	0.477–0.479	-0.209	0.480	0.002	0.476	0.483	0.479–0.481
		**R**	0.478	0.003	0.472	0.485	0.477–0.479	-0.209	0.480	0.002	0.477	0.482	0.479–0.481
	**PMI*y***	**L**	0.488	0.002	0.484	0.491	0.487–0.489	-0.102	0.489	0.001	0.486	0.491	0.489–0.490
		**R**	0.487	0.003	0.474	0.490	0.486–0.488	-0.205	0.489	0.001	0.486	0.492	0.489–0.490
	**PMI*z***	**L**	0.034	0.004	0.024	0.042	0.033–0.036	4.615	0.031	0.003	0.026	0.038	0.030–0.032
		**R**	0.035	0.001	0.026	0.044	0.033–0.036	6.061	0.031	0.002	0.026	0.037	0.030–0.031
**3rd**	**PMI*x***	**L**	0.477	0.003	0.470	0.484	0.476–0.478	-0.209	0.479	0.002	0.476	0.483	0.478–0.480
		**R**	0.477	0.003	0.469	0.483	0.476–0.478	-0.313	0.480	0.002	0.476	0.483	0.479–0.480
	**PMI*y***	**L**	0.489	0.002	0.486	0.492	0.488–0.489	-0.102	0.490	0.001	0.488	0.492	0.489–0.490
		**R**	0.488	0.002	0.485	0.492	0.488–0.489	-0.204	0.490	0.001	0.487	0.492	0.489–0.490
	**PMI*z***	**L**	0.034	0.004	0.024	0.044	0.033–0.036	4.615	0.031	0.003	0.027	0.035	0.030–0.032
		**R**	0.035	0.004	0.025	0.046	0.033–0.036	7.692	0.030	0.003	0.027	0.036	0.029–0.031
**4th**	**PMI*x***	**L**	0.479	0.003	0.473	0.486	0.478–0.480	-0.208	0.481	0.002	0.477	0.484	0.480–0.481
		**R**	0.478	0.003	0.472	0.484	0.477–0.479	-0.313	0.481	0.002	0.477	0.486	0.480–0.481
	**PMI*y***	**L**	0.487	0.002	0.484	0.492	0.487–0.488	-0.103	0.488	0.001	0.485	0.490	0.488–0.489
		**R**	0.487	0.002	0.483	0.491	0.486–0.488	-0.103	0.488	0.002	0.482	0.491	0.488–0.489
	**PMI*z***	**L**	0.034	0.004	0.023	0.041	0.032–0.036	4.615	0.031	0.003	0.026	0.037	0.030–0.032
		**R**	0.034	0.004	0.026	0.041	0.033–0.036	4.615	0.031	0.003	0.026	0.036	0.030–0.032
**5th**	**PMI*x***	**L**	0.484	0.003	0.479	0.491	0.483–0.485	-0.103	0.485	0.002	0.481	0.487	0.484–0.485
		**R**	0.484	0.002	0.480	0.489	0.483–0.485	-0.103	0.485	0.002	0.482	0.488	0.485–0.486
	**PMI*y***	**L**	0.475	0.004	0.468	0.487	0.474–0.476	-0.210	0.477	0.003	0.470	0.482	0.476–0.478
		**R**	0.476	0.004	0.468	0.484	0.474–0.477	-0.105	0.477	0.003	0.472	0.482	0.476–0.478
	**PMI*z***	**L**	0.041	0.006	0.022	0.051	0.039–0.043	3.797	0.038	0.004	0.032	0.049	0.037–0.040
		**R**	0.040	0.006	0.027	0.052	0.038–0.043	2.564	0.038	0.004	0.031	0.046	0.036–0.039

PAI, principal axes of inertia; SDI, sexual dimorphism index; 95% CI, 95% confidence interval of difference; PMIx, principal moments of inertia around the x axis of the bone; PMIy, principal moments of inertia around the y axis of the bone; PMIz, principal moments of inertia around the z axis of the bone.

All inertial variables were normalized by Equation (2).

**Tables 4**–**7** present independent sample *t*-test and Mann–Whitney U test results of eight variables of both sides of the investigated metatarsal bones between sexes. Highly significant sexual differences were found in SA: V of all metatarsals and in PMIs of the first to fourth metatarsals; in length of the first, third, and fourth metatarsals; in height of the first, fourth, and fifth metatarsals of both sides; and in width of the first metatarsal of the right side (p < 0.01). Sexual differences were found in length of the second and fifth metatarsals, in height of the third metatarsal, and in PMI*z* of the fifth metatarsal of both sides (p < 0.05). Sexual differences from the left side were found in width of the first metatarsal, in height of the second metatarsal, and in PMI*y* of the fifth metatarsal (p < 0.05). Significant differences from the right side were found in width of the fourth metatarsal and in PMI*x* and in bone density of the fifth metatarsal (p < 0.05). The first metatarsal was the most sexually dimorphic of five metatarsals, showing significant sexual differences of all variables except bone density, followed by the third metatarsal with statistical difference in bone height, length, SA: V, and PMIs.

**Table 4:** Independent sample *t*-test results of normalized length, width, and height of metatarsal bone *in vivo* based on its PAI between sexes.

Metatarsals	Geometric parameters		*t* value	*df*	*Sig. (2-tailed)*	95% CI	
						Lower	upper
**1st**	**Length**	**L**	-3.135	58.000	0.003**	-0.016	-0.004
		**R**	-4.319	58.000	0.000**	-0.020	-0.007
	**Height**	**L**	2.872	58.000	0.006**	0.002	0.010
		**R**	3.300	58.000	0.002**	0.003	0.011
**2nd**	**Length**	**L**	-2.411	50.345	0.020*	-0.014	-0.001
		**R**	-2.126	58.000	0.038*	-0.014	0.000
	**Width**	**L**	1.282	58.000	0.205	-0.001	0.006
		**R**	1.458	58.000	0.150	-0.001	0.007
	**Height**	**R**	1.881	58.000	0.065	0.000	0.009
**3rd**	**Length**	**L**	-3.079	58.000	0.003**	-0.016	-0.003
		**R**	-2.931	58.000	0.005**	-0.016	-0.003
	**Width**	**L**	1.853	58.000	0.069	0.000	0.007
		**R**	1.894	58.000	0.063	0.000	0.008
	**Height**	**L**	2.835	58.000	0.006**	0.002	0.010
		**R**	2.546	58.000	0.014*	0.001	0.011
**4th**	**Length**	**L**	-3.612	58.000	0.001**	-0.016	-0.005
		**R**	-3.780	58.000	0.000**	-0.018	-0.005
	**Width**	**R**	2.337	58.000	0.023*	0.001	0.008
	**Height**	**L**	3.333	58.000	0.002**	0.003	0.012
		**R**	3.326	58.000	0.002**	0.003	0.012
**5th**	**Length**	**R**	-2.510	58.000	0.015*	-0.014	-0.002
	**Width**	**L**	1.113	58.000	0.271	-0.002	0.007
	**Height**	**L**	3.330	58.000	0.002**	0.003	0.011
		**R**	3.089	58.000	0.003**	0.002	0.009

PAI, principal axes of inertia; 95% CI, 95% confidence interval of difference.

*Significance level: p < 0.05.

**Significance level: p < 0.01.

**Table 5:** Independent sample *t*-test results of SA: V (mm^-1^) and bone density (HU/1024) of metatarsal bone *in vivo* between sexes.

Metatarsals	Geometric parameters		*t* value	*df*	*Sig. (2-tailed)*	95% CI	
						Lower	upper
**1st**	**SA: V**	**L**	-8.432	58.000	0.000**	-0.041	-0.025
		**R**	-8.751	58.000	0.000**	-0.041	-0.026
	**Density**	**L**	1.694	58.000	0.096	-0.004	0.054
		**R**	1.270	58.000	0.209	-0.012	0.055
**2nd**	**SA: V**	**L**	-9.212	58.000	0.000**	-0.071	-0.045
		**R**	-10.189	58.000	0.000**	-0.074	-0.050
	**Density**	**L**	-1.930	58.000	0.058	-0.079	0.001
		**R**	-1.017	58.000	0.313	-0.060	0.020
**3rd**	**SA: V**	**L**	-12.808	58.000	0.000**	-0.074	-0.054
		**R**	-13.371	58.000	0.000**	-0.075	-0.056
	**Density**	**L**	-0.276	58.000	0.783	-0.043	0.032
		**R**	0.425	58.000	0.673	-0.028	0.044
**4th**	**SA: V**	**L**	-11.613	58.000	0.000**	-0.069	-0.049
		**R**	-12.038	52.206	0.000**	-0.073	-0.052
	**Density**	**L**	0.361	58.000	0.719	-0.030	0.043
**5th**	**SA: V**	**L**	-9.951	58.000	0.000**	-0.062	-0.041
		**R**	-10.264	58.000	0.000**	-0.063	-0.042
	**Density**	**L**	0.943	58.000	0.350	-0.018	0.050

95% CI, 95% confidence interval of difference.

*Significance level: p < 0.05.

**Significance level: p < 0.01.

**Table 6:** Independent sample *t*-test results of normalized PMIs of metatarsal bone *in vivo* based on its PAI between sexes.

Metatarsals	Geometric parameters		*t* value	*df*	*Sig. (2-tailed)*	95% CI	
						Lower	upper
**1st**	**PMI*x***	**L**	-3.482	47.595	0.001**	-0.006	-0.002
		**R**	-3.644	44.215	0.001**	-0.007	-0.002
	**PMI*y***	**L**	-4.276	58.000	0.000**	-0.008	-0.003
		**R**	-4.601	48.491	0.000**	-0.008	-0.003
	**PMI*z***	**L**	4.729	51.796	0.000**	0.005	0.013
		**R**	5.204	48.064	0.000**	0.006	0.014
**2nd**	**PMI*x***	**L**	-3.772	58.000	0.000**	-0.003	-0.001
		**R**	-4.669	46.392	0.000**	-0.004	-0.002
	**PMI*y***	**L**	-3.303	58.000	0.002**	-0.002	0.000
		**R**	-4.946	58.000	0.000**	-0.002	-0.001
	**PMI*z***	**L**	3.926	58.000	0.000**	0.002	0.005
		**R**	5.167	48.317	0.000**	0.003	0.006
**3rd**	**PMI*x***	**L**	-3.293	58.000	0.002**	-0.003	-0.001
		**R**	-4.692	58.000	0.000**	-0.004	-0.002
	**PMI*y***	**L**	-3.693	43.838	0.001**	-0.002	-0.001
		**R**	-4.217	45.266	0.000**	-0.002	-0.001
	**PMI*z***	**L**	3.710	58.000	0.000**	0.002	0.005
		**R**	4.854	46.054	0.000**	0.003	0.006
**4th**	**PMI*x***	**L**	-3.457	46.444	0.001**	-0.003	-0.001
		**R**	-4.448	58.000	0.000**	-0.004	-0.002
	**PMI*z***	**L**	3.365	47.199	0.002**	0.001	0.005
		**R**	4.148	50.493	0.000**	0.002	0.006
**5th**	**PMI*x***	**L**	-1.652	49.618	0.105	-0.002	0.000
		**R**	-2.312	50.937	0.025*	-0.002	0.000
	**PMI*y***	**L**	-2.088	58.000	0.041*	-0.004	0.000
		**R**	-1.823	50.929	0.074	-0.003	0.000
	**PMI*z***	**R**	2.126	50.237	0.038*	0.000	0.005

PAI, principal axes of inertia; 95% CI, 95% confidence interval of difference.

*Significance level: p < 0.05.

**Significance level: p < 0.01.

**Table 7:** Mann-Whitney U test results of variables of metatarsal bone *in vivo* based on its PAI between sexes.

Sides	Geometric parameters	Mann–Whitney U	Wilcoxon W	*Z*	*Sig.*
**Left**	**1st width**	282	747	-2.484	0.013*
	**2nd height**	278	743	-2.543	0.011*
	**4th width**	329	794	-1.789	0.074
	**4th PMI*y***	626	1091	2.602	0.009**
	**5th length**	647	1112	2.913	0.004**
	**5th PMI*z***	298	763	-2.247	0.025*
**Right**	**1st width**	188	653	-3.874	0.000**
	**4th PMI*y***	625	1090	2.587	0.010*
	**4th density**	350	815	-1.478	0.139
	**5th density**	290	755	-2.366	0.018*
	**5th width**	367	832	-1.227	0.220

PAI, principal axes of inertia.

*Significance level: p < 0.05.

**Significance level: p < 0.01.

The subjects’ body height and weight showed low correlations (|*r_s_* | < 0.40) with variables except SA: V (0.60 <|*r_s_* | < 0.81). Length presented multicollinearity with some variables (|*r_p_* | > 0.80), and PMI*x*, PMI*y* showed a high correlation with PMI*z* (|*r_p_* | > 0.80) (**Supplementary File III**; **Tables S6**–**S15**). Considering the high SDI and significant sexual difference by the independent *t*-test, the width, height, PMI*z*, bone density, and SA: V were selected as independent variables for the SDFA. The probability of variables was more than 0.001, indicating the absence of outlier in the samples. Two multivariate outliers were identified and removed in our study, which were found in the first metatarsal bone of the left and right sides, respectively. The homogeneity of variance matrices was evaluated by Box’s M with p > 0.001 for both sides in our analysis (**Supplementary File III**; **Table S16**). The multivariate normality of variables was found in the first metatarsal of the left side and in the second to fourth metatarsals of the right side (**Supplementary File III**; **Table S17**). Linearity among five variables was presented in matrix scatter plots (**Supplementary File III**; **Figures S5A, B**).

**Tables 8A**, **B** describe the SDFA results for sex determination. The results of standardized canonical discriminant coefficients and structure matrix show that SA: V has the highest correlation with discriminant functions and thus contributes most to sex estimation.

**Table 8A:** Stepwise discriminant function analysis for left metatarsal bone.

Functions	Wilk’s lambda			Unstandardized coefficients*^c^*	Structure matrix*^d^*	Standardized coefficients*^e^*	Group centroids*^f^*		Sectioning point*^g^*
	Wilk’s lambda*^a^*	Chi-square	sig*^b^*				Male	Female	
Function 1 Measurements of the 1st metatarsal bone									
SA: V	0.283	69.969	0.000	-72.779	-0.686	-1.098	1.536	-1.589	-0.027
Bone density				13.129	0.144	0.745		
Height				64.683	0.278	0.504		
(constant)				-13.556				
Function 2 Measurements of the 2nd metatarsal bone									
SA: V	0.380	55.225	0.000	39.926	0.946	0.974	-1.257	1.257	0.000
Height				-35.974	-0.242	-0.325		
(constant)				-8.664				
Function 3 Measurements of the 3rd metatarsal bone									
SA: V	0.241	81.189	0.000	51.104	0.947	0.984	-1.746	1.746	0.000
Height				-38.990	-0.210	-0.324		
(constant)				-13.755				
Function 4 Measurements of the 4th metatarsal bone									
SA: V	0.301	69.087	0.000	50.593	1.000	1.000	-1.499	1.499	0.000
(constant)				-20.593				
Function 5 Measurements of the 5th metatarsal bone									
SA: V	0.345	60.676	0.000	51.174	0.948	1.024	-1.355	1.355	0.000
Bone density				-4.971	-0.090	-0.327		
(constant)				-10.718				

^a^At each step, the variable that minimizes the overall Wilks’ lambda is entered. Minimum partial F to enter is 3.84. Maximum partial F to remove is 2.71.

^b^p value is 0.000, which means the significant level at p < 0.001.

^c^Unstandardized canonical discriminant functions evaluated at group means. Take Function 1 for example, Y = 13.129 * bone density + 64.683 * height - 72.779 * SA: V - 13.556.

^d^Structure matrix indicates the pooled within-group correlations between discriminating variables and standardized canonical discriminant functions.

^e^Standardized coefficients represent the contribution of the variable to sex discrimination.

^f^Unstandardized canonical discriminant functions evaluated at group means.

^g^When the group mean of male is positive, discriminant score (Y) > sectioning point would be considered as male; while the group mean of male is negative, discriminant score (Y) < sectioning point would be considered as male.

**Table 8B:** Stepwise discriminant function analysis for right metatarsal bone.

Functions	Wilk’s lambda			Unstandardized coefficients	Structure matrix	Standardized coefficients	Group centroids		Sectioning point
	Wilk’s lambda	Chi-square	sig				Male	Female	
Function 1 Measurements of the 1st metatarsal bone									
SA: V	0.293	68.207	0.000	-61.716	-0.728	-0.911	1.554	-1.503	0.026
PMI*z*				77.849	0.504	0.535		
Bone density				8.292	0.127	0.530		
(constant)				-2.848				
Function 2 Measurements of the 2nd metatarsal bone									
SA: V	0.317	65.407	0.000	37.552	0.912	0.888	-1.442	1.442	0.000
PMI*z*				-124.899	-0.463	-0.410		
(constant)				-10.559				
Function 3 Measurements of the 3rd metatarsal bone									
SA: V	0.224	85.167	0.000	52.538	0.944	0.995	-1.828	1.828	0.000
Height				-35.799	-0.180	-0.333		
(constant)				-14.859				
Function 4 Measurements of the 4th metatarsal bone									
SA: V	0.213	87.386	0.000	54.196	0.822	1.087	-1.890	1.890	0.000
Bone density				-8.403	-0.058	-0.528		
Height				-37.812	-0.227	-0.333		
(constant)				-1.557				
Function 5 Measurements of the 5th metatarsal bone									
SA: V	0.277	73.259	0.000	55.060	0.833	1.092	-1.590	1.590	0.000
Bone density				-9.850	-0.147	-0.610		
(constant)				-4.021				

The accuracies of discriminant functions based on original samples and cross-validated samples are reported in **Tables 9A**, **B**. The sex determination accuracies of the original samples were between 88.3% and 98.3% of both sides. Moreover, the percentage of correct classification of cross-validated samples was also between 88.3% and 98.3% of both sides. No significant variance was observed in accuracies between the original samples and the cross-validated samples, revealing the steady predication ability of the discriminant functions. The highest accuracy was found in the third and fourth metatarsal bone of the right side—98.3% in our cases. The accuracy of the right side was slightly higher than that of the left side.

**Table 9A:** Accuracy of classification results of the original and cross-validated samples (left side)*^a^*.

Functions	Male		Female		Total average (%)
	N	%	N	%	
**Function 1**					
**Original**	27/30	90.0	27/29	93.1	91.5
**Cross-validated**	27/30	90.0	27/29	93.1	91.5
**Function 2**					
**Original**	27/30	90.0	26/30	86.7	88.3
**Cross-validated**	27/30	90.0	26/30	86.7	88.3
**Function 3**					
**Original**	29/30	96.7	29/30	96.7	96.7
**Cross-validated**	29/30	96.7	28/30	93.3	95.0
**Function 4**					
**Original**	29/30	96.7	26/30	86.7	91.7
**Cross-validated**	29/30	96.7	26/30	86.7	91.7
**Function 5**					
**Original**	29/30	96.7	26/30	86.7	91.7
**Cross-validated**	29/30	96.7	25/30	83.3	90.0

^a^Cross-validation is done only for those cases in the analysis. In cross-validation, each case is classified by the functions derived from all cases other than those cases.

**Table 9B:** Accuracy of classification results of the original and cross-validated samples (right side).

Functions	Male		Female		Total average (%)
	N	%	N	%	
**Function 1**					
**Original**	26/29	89.7	28/30	93.3	91.5
**Cross-validated**	26/29	89.7	28/30	93.3	91.5
**Function 2**					
**Original**	29/30	96.7	26/30	86.7	91.7
**Cross-validated**	29/30	96.7	26/30	86.7	91.7
**Function 3**					
**Original**	30/30	100.0	29/30	96.7	98.3
**Cross-validated**	30/30	100.0	29/30	96.7	98.3
**Function 4**					
**Original**	30/30	100.0	29/30	96.7	98.3
**Cross-validated**	30/30	100.0	29/30	96.7	98.3
**Function 5**					
**Original**	30/30	100.0	27/30	90.0	95.0
**Cross-validated**	30/30	100.0	27/30	90.0	95.0

## 4 Discussion

Parameters such as the voltage, parameter of field of view, and reconstruction settings influence the accuracy of measurements (length, width, height, surface area, and volume) during the 3D reconstruction of bone. The ICC values of bone length, width, and height presented high consistency of metatarsal measurements between two reconstructions while the ICC values of surface area and volume were lower than those of linear measurements. Volume is a higher-order quantity compared to length, width, and height. For example, the ICC value of the side length of a square is 0.91, while that of its volume is 0.75. The ICC value of SA: V was also calculated, and the lowest ICC values rose to 0.93, indicating that SA: V has higher consistency than surface area and volume. Therefore, SA: V instead of surface area and volume was used in this study. Parameters such as field of view and voltage were not set the same in the two scans, which also affected the ICC values of the reconstructed metatarsal measurements. From this view, the ICC values of metatarsals’ measurements between two reconstructions were high, indicating that the reconstructed 3D bone model was precise under these reconstruction parameter settings. It is reasonable to assume that the parameter setting mentioned in *Methods* generated an accurate reconstruction model.

The sexual dimorphism in the human skeleton system is well studied (10, 39–46). Researchers keep on exploring the potential of bones in sex estimation, for instance, mandible (47), long bones of the upper limb (44, 46, 48), metacarpals and phalanges (45), pelvis (41, 42), tibia (9), metatarsal (19, 49, 50), and proximal foot phalanges (51). The accuracy of sex estimation provided by different parts of the bone varied ranged from 66% to 99%. For metatarsal bone, this study showed for the first time that the SA: V and PMIz of metatarsal bone with significant sexual dimorphism may be successfully implemented for sex estimation. The discriminant accuracy of metatarsal’s geometric and inertial variables of Chinese samples in this study were between 88.3% and 98.3%, which were comparable to the accuracy reported in the Portuguese Caucasian population (83.0%–100.0%) (49), the Greek samples (80.7%–90.1%) (17), the Iranian population (82.6%–86.9%) (50), and the Egyptian population (81.3%–97.5%) (52). The accuracy variation can be found in different populations, indicating that the classification accuracy of metatarsal bone was population-specific. Gibelli et al. reported the superiority of linear measurements over volumetric measurements in sex estimation (19). In our study, the SA: V and PMIz of five metatarsal bones showed greater sexual dimorphism than linear variables and SA: V contributes most to sex estimation. The discriminant function based on SA: V provided 91.7% accuracy (91.7% cross-validated). Studies found that SA: V would decrease with increasing body size as trabeculae became thicker (53–55). In our cases, high and negative correlations (0.60 <|*r_s_* | < 0.81) were found between SA: V and body weight as well as between SA: V and body height, indicating that high classification accuracy and significant sexual dimorphism of the SA: V value may arise from the differences in body size between males and females. On the other hand, normalized linear and inertial variables presented low correlations (|*r_s_* | < 0.4) with body height and weight, suggesting that normalized bone height and PMI*z* value were less likely affected by the differences in individuals’ height and weight in sex estimation.

It is known from the natural principles that form follows the function. Loading can significantly modify bone shape and mass, and this influence is long-lasting (56). Bone robusticity is generally considered as an important indicator of the magnitude and nature of the force that acts on the bone, providing information about the habitual behavior of organism (57–59). Some studies reported the sexual differences in robusticity of hand and foot bones (17, 51, 60). In our study, high SDIs of bone height and PMI*z* may reflect differences in genetics and physical activity level between sexes. In linear measurements, higher SDI values were found in bone height other than in bone width and length, which was consistent with findings of the literature (17, 50). Ruff et al. found that the diaphyseal cross-sectional size changed significantly with the increase of mechanical load (body weight increase) (61). Similar results were discovered by Lieberman et al., reporting a significant change in the diaphyseal cross-sectional geometry of limb bone of sheep after 3 months’ moderate exercise (62). PMI is associated with bone morphology and mass distribution. Significantly higher PMI*z* values in the male than in female population presumably come as a consequence of the increased physical activities of the male subjects. Namely, tarsals and metatarsals, with ligaments and tendons from the foot arch, could resist impact and maintain stability during walking, running, or jumping. Metatarsals mainly bear the longitudinal pressure from its base to head, which explains the greatest variation in ratio of PMI*z* values—the load bearing along the *z* axis has presumably undergone adaptability changes (63). The effect of age on bone dimension and robusticity should be noted. No consensus has been reached. It was found that the subperiosteal diameter increased with age (64–66). However, some studies found that mechanical loads during adulthood had little effect on the external dimensions of long bone diaphysis and the age-related changes in diaphyseal cross-sectional size of bone were not evident (67–70). It may indicate that the diaphyseal cross-sectional properties of bone were mainly affected by physical activity before skeletal maturity (70). The effect of age and mechanical loads on the geometric and inertial characteristics of metatarsal bone needs further in-depth studies of larger sample size of different ethnical/racial groups.

How to best implement the CSI analysis for sex estimation in forensics and archeology can be discussed, but some methodological issues have to be resolved. Firstly, the measurements that differ in the male and female populations should be identified. Those variables may be geometric (length, width, height, SA: V) and inertial (PMIs). The present paper paves the way for sex estimation by introducing the concept of PMI-oriented bone coordinate system normalization. PMI*z* is the attribute of the rotational movements; it is an analog of the mass of the translational movements. The advantage of the inertial analysis is the evidence that such analysis does not depend on factors such as nutrition and genetics, as is the case for linear variables. It quantitatively assesses the foot bone physical properties, providing more accurate data than qualitatively measured pelvis and cranium or metrical approaches focusing on a single bone element (20, 71). Secondly, the method that yields precise measurements based on 3D models should be determined. The present paper introduces a bone positioning method. The body coordinate system sets COM as the origin, achieving bone location, and sets three PAIs of bone as the body coordinate axes, positioning bone posture to avoid measurement error caused by different scanning positions, which ensures the high accuracy of dimensions along the axis (bone length, width, and height). One additional advantage of this method is that the dimension of bone along the axis can be obtained automatically, reducing the possible error caused by manual measurement.

This study is the continuation of the ongoing scientific efforts to employ virtual 3D reconstruction in determination of individuals’ sex and age (2, 72). Foot bones were chosen purposely due to their wide availability in both archeological and forensic context owing to more resistance to the rigors of time than long bones (18). The accuracy of virtual analysis of the metatarsal bone is proven in a previous investigation that evaluated the efficacy of a radiological method to estimate the individuals’ sex using measurements of the first and second metatarsals of a Portuguese Caucasian population (49).

The high prevalence of metatarsal bones at archeological and forensic sites justifies that the proposed method may be widely applied in archeology and forensics. The wide application of 3D CSI in forensics is constrained because of ethical issues, which also has limited the establishment of populations’ databases. However, the data obtained during routine medical examination may be stored and subsequently employed in forensic analysis. The analysis of metatarsal bones using radiography is rapid and noninvasive. The advantages of 3D CSI forensic analysis include, besides the potential for sex estimation, precise documentation and 3D demonstration of forensic findings for the court, reduction of trauma, and decreased risk of transmission of disease (1). It is interesting to note that the CSI analysis of the fourth metatarsal even allowed the scientists to explain the ground-dwelling biped walking pattern of *Australopithecus afarensis* dating back to 3.2 million years ago (73).

Some weaknesses of virtopsy-oriented skeletal assessments should be noted though. The quality of the CSI can be affected by many factors, including the scanning posture, error aggregation, resolution, and dose, resulting in the inconsistency in the 3D reconstruction models (74–76). Studies have shown that 3D bone models can achieve high accuracy at the sub-millimeter scale, while increasing the voxel resolution (from 0.3 to 0.15 mm) does not improve the accuracy of the models (77). Our previous study (33) compared the accuracy of 3D bone models reconstructed with different anisotropic voxels (different pixel sizes) and found no significant differences in the linear, volume, and surface area measurements of the models. In particular, the linear measurement values remained highly consistent, indicating that pixel size had no significant influence on model accuracy at the submillimeter scale. Micro CT scanning, providing scans at the few-micron level for small size samples, is commonly used to evaluate the trabecular bone microstructure (78–80). However, the effects of multi-detector CT and micro CT with different resolutions on the accuracy of 3D bone models are still unclear and need to be further studied. The standardization of the body coordinate system of bone is able to avoid the adverse effects arising from different scanning postures, while rating those quantities by percentage could reduce the effect from resolution and dose. The same bone can be reconstructed by a different operator or it can be reconstructed by the same operator for many times, so the parameter setting of the reconstruction process should be taken into consideration when comparing the results of different studies on the CSI analysis of the skeletal tissue. For statistical results, some assumptions were not confirmed, such as multivariate normality of the second to fifth metatarsals of the left side and the first and fifth metatarsals of the right side. The accuracy of formulae based on these metatarsal variables should be treated with caution, although discriminant function analysis is relatively robust against deviations from multivariate normality (81). It should be further highlighted that the accuracy of estimation may be influenced by the characteristics of the selected bone, population, sample size, and age. The promising results (cross-validated accuracy ranges) of this study may in part be driven by the small sample size as well as the same young age-group. The potential future application of this new proposed method for sex determination on unidentified individuals would not be as accurate as suggested in this study. Therefore, the methods should be tested (or independently developed) for distinct population groups, before being widely applied in individuals of unknown population origin (i.e., unidentified skeletons in forensic and bioarcheological contexts).

## 5 Conclusion

This study demonstrates that sexual dimorphism was found in both metatarsal bones’ geometric and inertial variables. A profound analysis of 60 subjects’ metatarsals revealed that discriminant functions based on geometric and inertial variables of metatarsal bones generated accuracies of 88.3%–98.3% in sex estimation. The ongoing studies are under way to test the potential of the proposed method on the sex determination of archaeological remains and of larger sample size with different population groups.

## Data Availability Statement

The raw data supporting the conclusions of this article will be made available by the authors, without undue reservation.

## Ethics Statement

The studies involving human participants were reviewed and approved by the Ethical Committee of Fujian Normal University. The patients/participants provided their written informed consent to participate in this study.

## Author Contributions

YFF and MD conceived the study. YL, DA, RL, YXF, KD, MM, and GY collected and analyzed the data. YFF, YL, DA, and ZL wrote the manuscript, and all authors revised the final manuscript.

## Funding

This study was supported by the National Natural Science Foundation of China (grant numbers 11672075, 11972119), Natural Science Foundation of Fujian Province (grant number 2019J01429), and Ministry of Education, Science and Technological Development of the Republic of Serbia (grant number III 45005).

## Conflict of Interest

The authors declare that the research was conducted in the absence of any commercial or financial relationships that could be construed as a potential conflict of interest.

## Publisher’s Note

All claims expressed in this article are solely those of the authors and do not necessarily represent those of their affiliated organizations, or those of the publisher, the editors and the reviewers. Any product that may be evaluated in this article, or claim that may be made by its manufacturer, is not guaranteed or endorsed by the publisher.

## Abbreviations

3D, three-dimensional; CSI, cross-section imaging; HU, Hounsfield units; PMI, principal moment of inertia; PAI, principal axis of inertia; SDI, sexual dimorphism index; ICC, intraclass correlation coefficient; SA: V, surface-area-to-volume ratio; SDFA, stepwise discriminant function analysis.
