# Supplementary material for: Study of Sexual Dimorphism in Metatarsal Bones: Geometric and Inertial Analysis of the Three-Dimensional Reconstructed Models
Source: Front Endocrinol (Lausanne). 2021 Oct 14;12:734362. doi: 10.3389/fendo.2021.734362 (PMC8551807; doi:10.3389/fendo.2021.734362)
Supplement: Supplementary file 1 [file DataSheet_1.pdf]

Supplementary File I

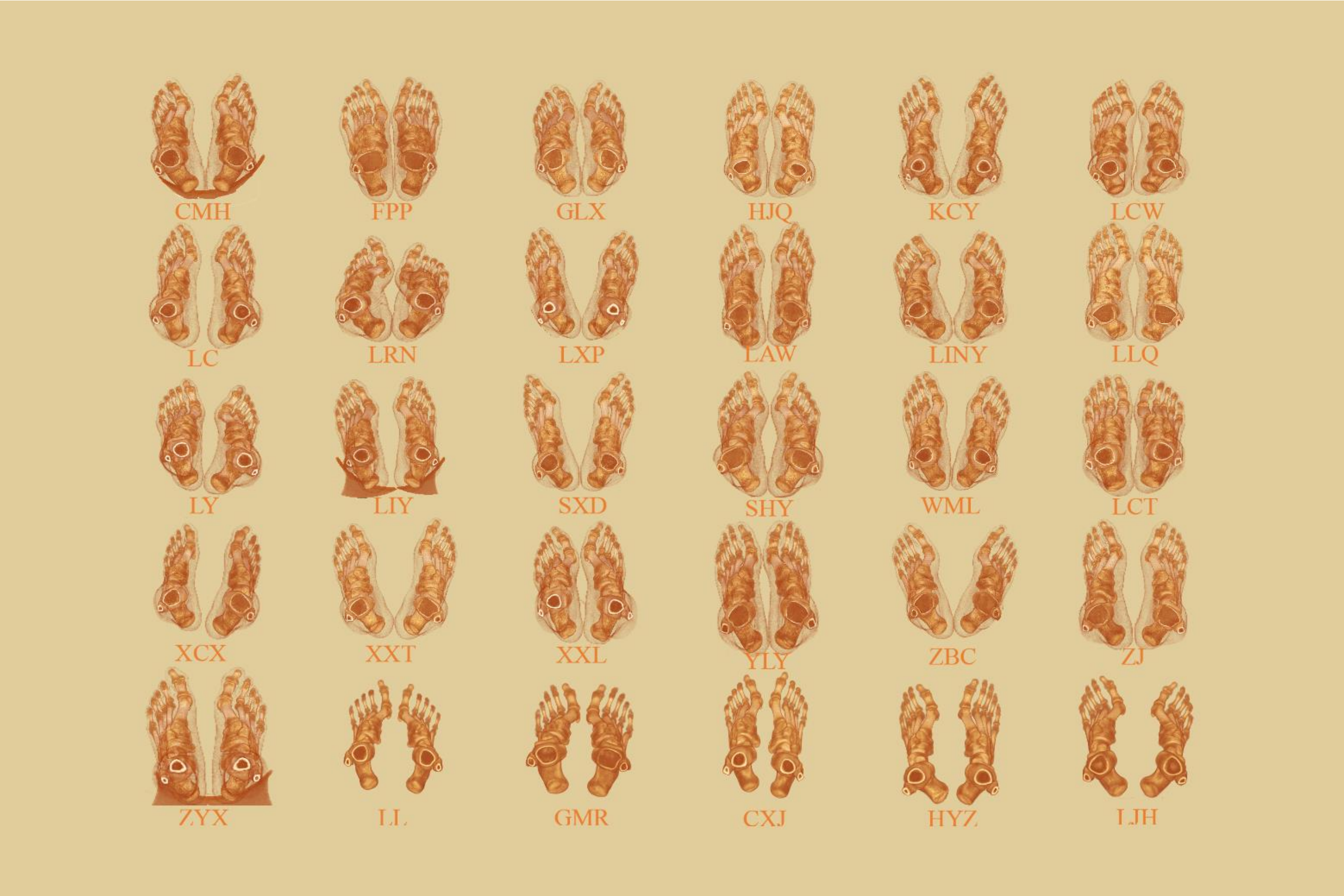

Figure S1-A Scanning posture of 30 female subjects. Capital letters are the initials of the subjects.

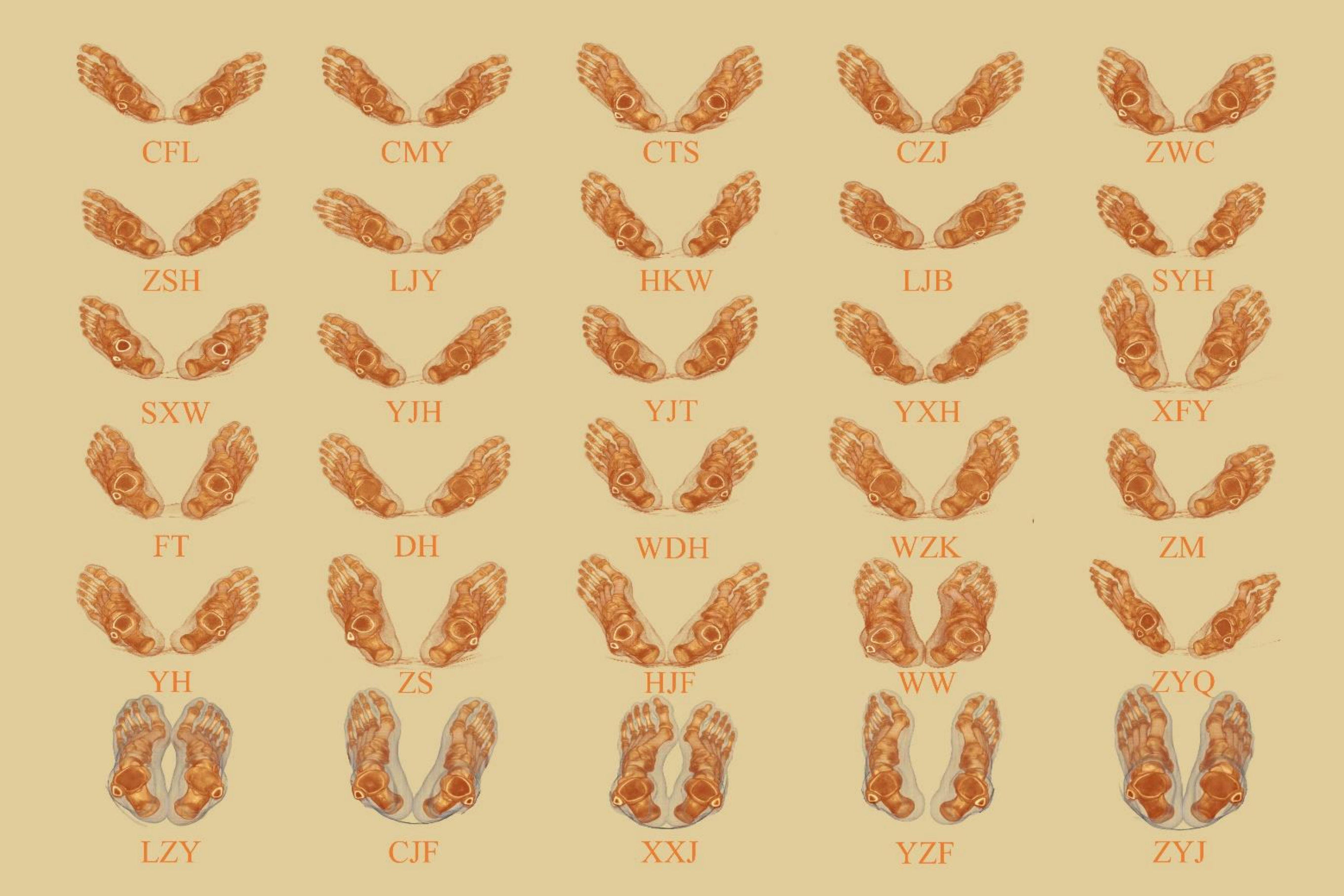

Figure S1-B Scanning posture of 30 male subjects. Capital letters are the initials of the subjects.

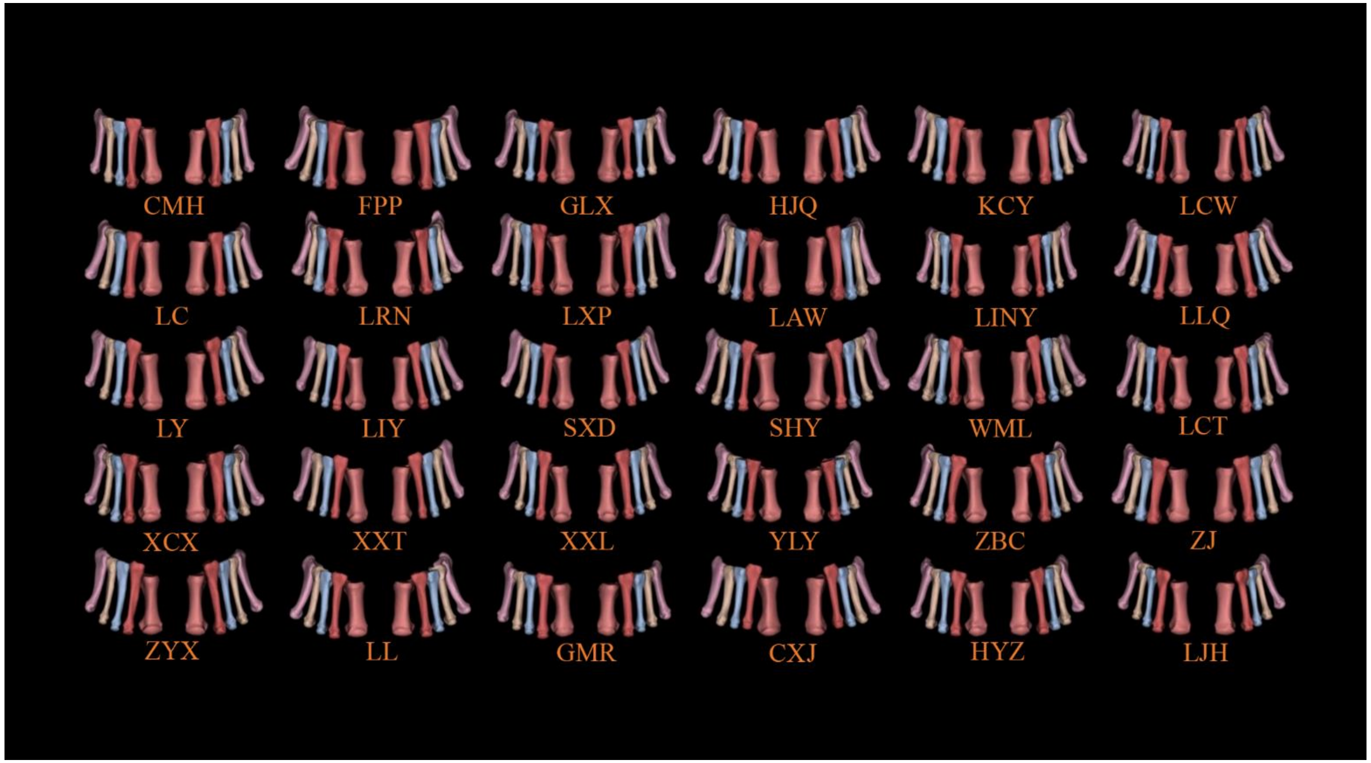

Figure S2-A 300 reconstructed 1st - 5th metatarsal bones of 30 female subjects. Capital letters are the initials of the subjects.

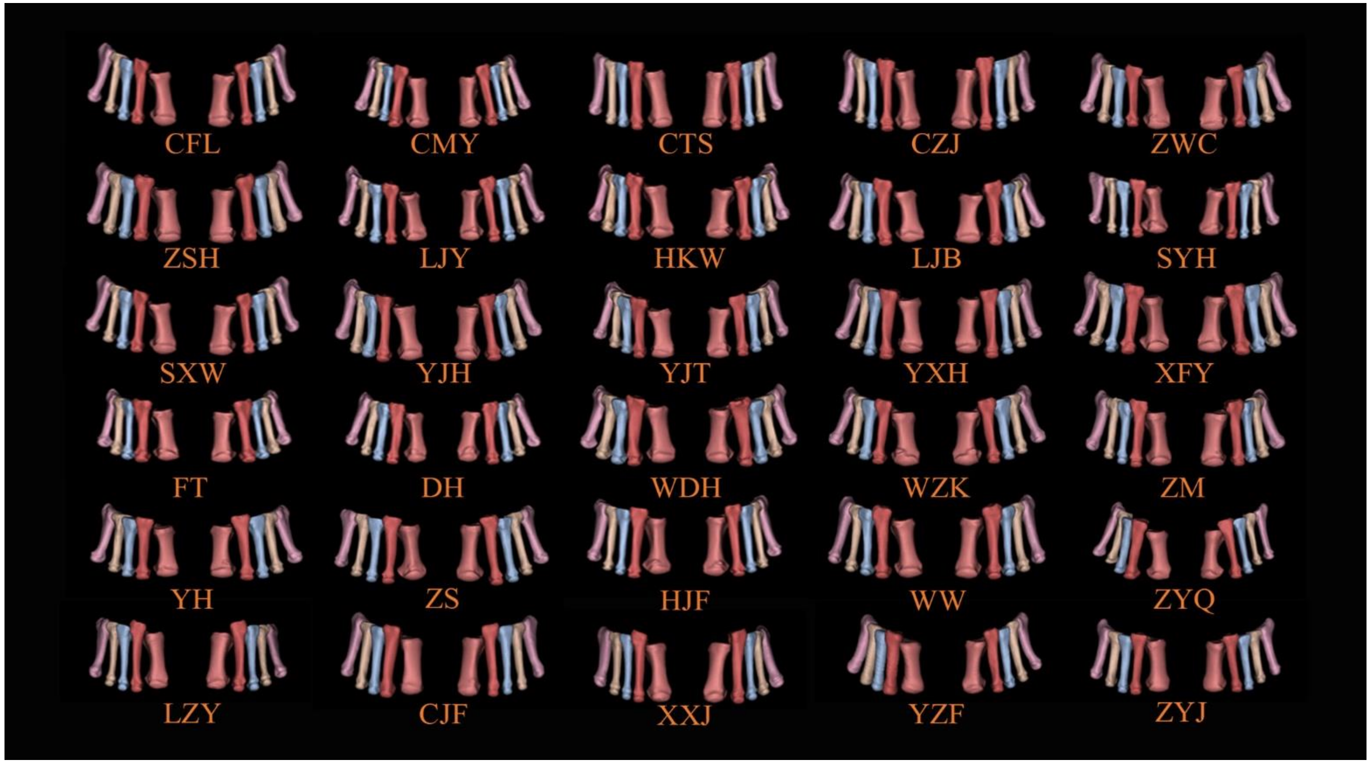

Figure S2-B 300 reconstructed 1st - 5th metatarsal bones of 30 male subjects. Capital letters are the initials of the subjects.

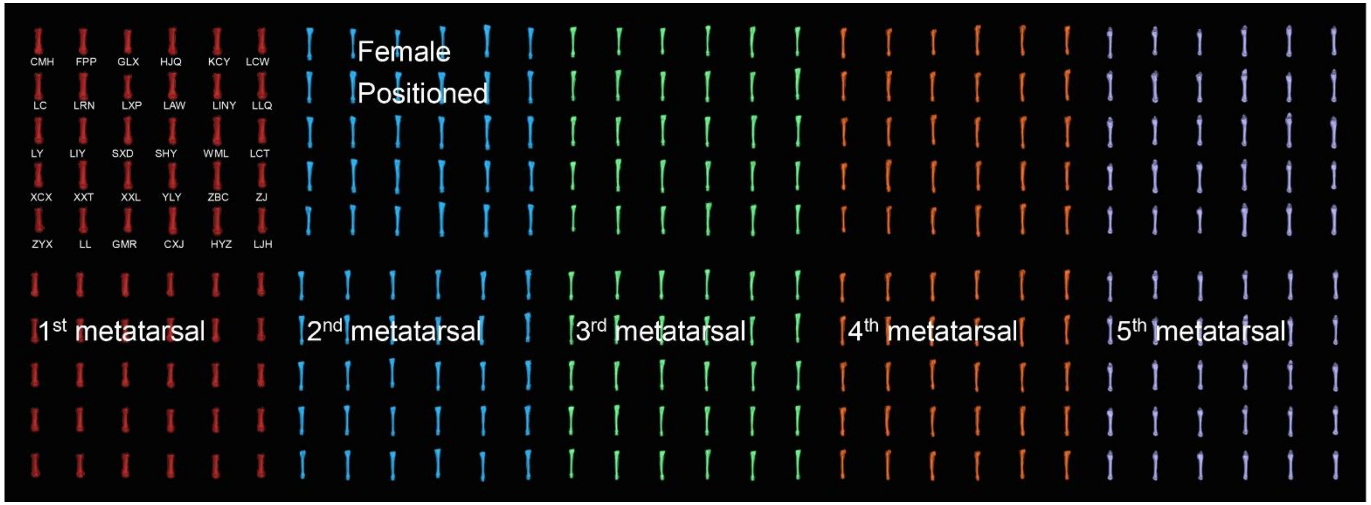

Figure S3-A 300 pieces of positioned 1st - 5th metatarsal bones of 30 female subjects. Capital letters are the initials of the subjects.

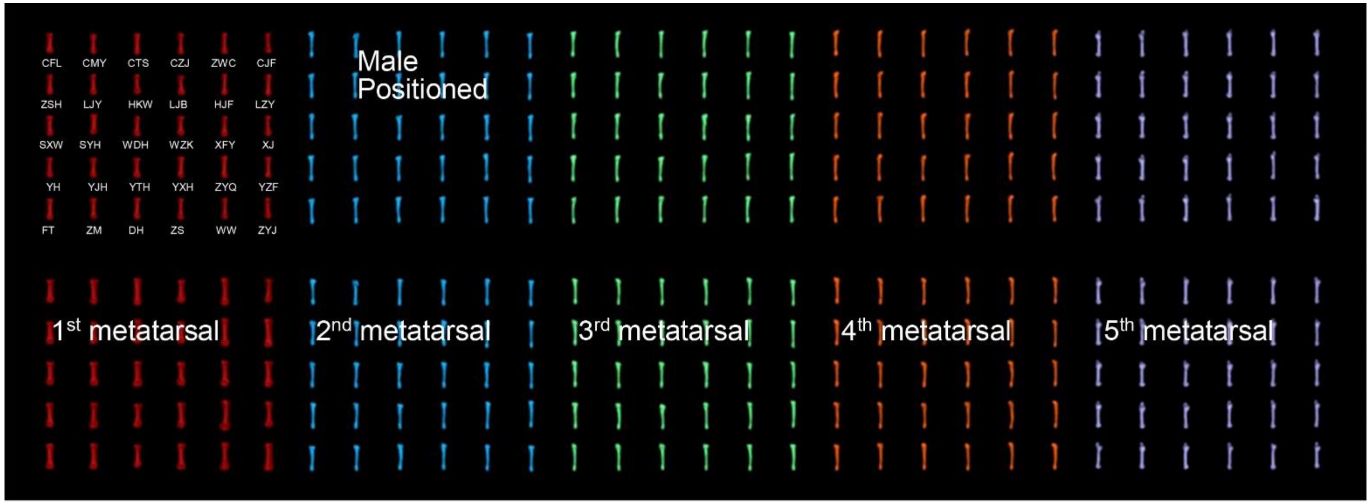

Figure S3-B 300 pieces of positioned 1st - 5th metatarsal bones of 30 male subjects. Capital letters are the initials of the subjects.

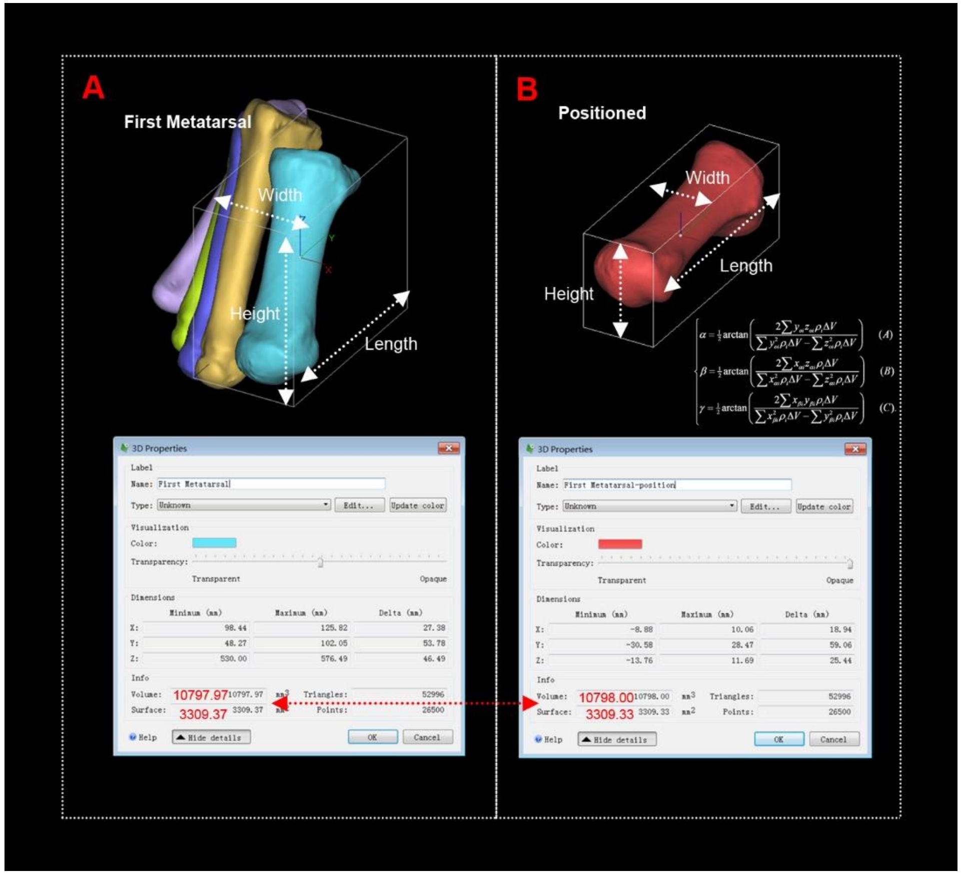

Figure S4 Reconstructing and positioning the metatarsal bone. (A) Length, width, height, volume and surface area of the reconstructed 1st metatarsal bone. (B) Length, width, height, volume and surface area of the positioned 1st metatarsal bone. The positioning method was from Fan et al., 2012.

Reference:

Fan, Y., Fan, Y., Li, Z., Lv, C., and Zhang, B. (2012). Bone surface mapping method. PloS one, 7(3), e32926.
