# Supplementary material for: Study of Sexual Dimorphism in Metatarsal Bones: Geometric and Inertial Analysis of the Three-Dimensional Reconstructed Models
Source: Front Endocrinol (Lausanne). 2021 Oct 14;12:734362. doi: 10.3389/fendo.2021.734362 (PMC8551807; doi:10.3389/fendo.2021.734362)
Supplement: Supplementary file 2 [file DataSheet_2.pdf]

1 Principal axes of inertia (PAI) and moments of inertia (MI) of the cross-sectional imaging (CSI)

We use oxy to show the rectangular coordinate system of the CSI's COM. The CSI is consisted of finite surface area element of volume (dV). I_x, I_y stand for moments of inertia (MI) on axis x, y respectively, and I_{xy} stands for product of inertia (PI), which is expressed by the equation:

$$\begin{cases} I_x = \int y^2 \rho dA & (a) \\ I_y = \int x^2 \rho dA & (b) \\ I_{xy} = \int xy\rho dA & (c) \end{cases} \qquad (1)$$

where dA stands for area element, ρ for its grey value, and (x, y) for its position coordinate.

Let the coordinate system rotate φ around the CSI's COM. A new coordinate system will be formed: $ox_\varphi y_\varphi$. The relation between surface area element coordinates (x_φ, y_φ) and those of (x, y) is:

$$\begin{cases} x_\varphi = x\cos\varphi + y\sin\varphi & (a) \\ y_\varphi = -x\sin\varphi + y\cos\varphi & (b) \end{cases} \qquad (2)$$

I_x^α, I_y^α stand for the MI of axis x_φ, y_φ respectively, which is expressed as:

$$\begin{cases} I_x^\varphi = \int y_\varphi^2 \rho dA & (a) \\ I_x^\varphi = \int x_\varphi^2 \rho dA & (b) \end{cases} \qquad (3)$$

Substitute Eq 2(b) into 3(a), we will get:

$$\begin{aligned} I_x^\varphi &= \int (-x\sin\varphi + y\cos\varphi)^2 \rho dA \\ &= \int \left(x^2\sin^2\varphi - 2xy\sin\varphi\cos\varphi + y^2\cos^2\varphi\right)\rho dA \end{aligned} \qquad (4)$$

Substitute 2(a) into 3(b), we will get:

$$\begin{aligned} I_y^\varphi &= \int (x\cos\varphi + y\sin\varphi)^2 \rho dA \\ &= \int \left(x^2\cos^2\varphi + 2xy\sin\varphi\cos\varphi + y^2\sin^2\varphi\right)\rho dA \end{aligned} \qquad (5)$$

Add Eq 4 and Eq 5, we will get:

$$\begin{aligned} I_x^\varphi + I_y^\varphi &= \int \left(x^2\left(\sin^2\varphi + \cos^2\varphi\right) + y^2\left(\cos^2\varphi + \sin^2\varphi\right)\right)\rho dA \\ &= \int \left(x^2 + y^2\right)\rho dA \\ &= I_x + I_y \end{aligned} \qquad (6)$$

Eq 6 shows that when CSI rotates around its COM, its MI is invariable, which means Eq 6 is indeterminate. To indeterminate equation, we can set up an equation as follows:

$$f(\alpha)=I_x^{\varphi}-I_y^{\varphi} \qquad (7)$$

Substitute Eq 4 and Eq 5 into Eq 7, we will get:

$$f(\alpha)=\int\left(x^2\left(\sin^2\varphi-\cos^2\varphi\right)-4xy\sin\varphi\cos\varphi-y^2\left(\sin^2\varphi-\cos^2\varphi\right)\right)\rho dA \quad (8)$$

Since $2\sin\varphi\cos\varphi=\sin 2\varphi$, $\cos^2\varphi-\sin^2\varphi=\cos 2\varphi$, substitute these relations into Eq 8, we will get:

$$f(\varphi)=\int\left(-x^2\cos 2\varphi-2xy\sin 2\varphi+y^2\cos 2\varphi\right)\rho dA \qquad (9)$$

Let

$$\frac{df(\varphi)}{d\varphi}=0,$$

and we will get:

$$\frac{df(\varphi)}{d\varphi}=\int\left(2x^2\sin 2\varphi-4xy\cos 2\varphi-2y^2\sin 2\varphi\right)\rho dA=0 \qquad (10)$$

By Eq 10, we will get:

$$2\sin 2\varphi\int x^2\rho dA-4\cos 2\varphi\int xy\rho dA-2\sin 2\varphi\int y^2\rho dA=0 \qquad (11)$$

By Eq 1 and Eq 11, we will get:

$$2\sin 2\varphi I_y-4\cos 2\varphi I_{xy}-2\sin 2\varphi I_x=0 \qquad (12)$$

Divide both sides of Eq 12 by $2\cos 2\varphi$, and we will get:

$$\tan 2\varphi=-\frac{2I_{xy}}{I_x-I_y}$$

The inverse function of Tangent is:

$$\varphi=-\frac{1}{2}\arctan\left(\frac{2I_{xy}}{I_x-I_y}\right) \qquad (13)$$

Eq 13 shows that by only one rotation, we can position the CSI.

2 PAI and MI of the reconstruction of CSI

2.1 To rotate around axis *x*

We use $oxyz$ to stand for the spatial rectangular coordinate system of the COM of the reconstructed CSI, which was consisted of finite elements of volume. I_x, I_y, I_z stand for the MI

of axis x, y, z respectively.

Their MI is:

$$\begin{cases} I_x = \int \left(y^2 + z^2\right)\rho dV \\ I_y = \int \left(x^2 + z^2\right)\rho dV \\ I_z = \int \left(x^2 + y^2\right)\rho dV \end{cases} \quad (14)$$

Their PI is:

$$\begin{cases} I_{xy} = \int xy\rho dV \\ I_{yz} = \int yz\rho dV \\ I_{xz} = \int xz\rho dV \end{cases} \quad (15)$$

where dV stands for element of volume, ρ for its grey value, and (x, y, z) for its position coordinate.

Let the body coordinate system of the reconstructed CSI's COM rotate α around *x*. Then a new coordinate system of $ox_\alpha y_\alpha z_\alpha$ will be formed. The relation between body element of volume coordinate system of $(x_\alpha, y_\alpha, z_\alpha)$ and (x, y, z) will be:

$$\begin{cases} x_\alpha = x & (a) \\ y_\alpha = y\cos\alpha - z\sin\alpha & (b) \\ z_\alpha = y\sin\alpha + z\cos\alpha & (c) \end{cases} \quad (16)$$

Substitute Eq 16(b) and 16(c) into the MI of $I_x^\alpha = \int \left(y_\alpha^2 + z_\alpha^2\right)\rho dV$ relative to axis *x*, and we will get:

$$\begin{aligned} I_x^\alpha &= \int \left((y\cos\alpha - z\sin\alpha)^2 + (y\sin\alpha + z\cos\alpha)^2\right)\rho dV \\ &= \int \left(y^2\cos^2\alpha - 2yz\cos\alpha\sin\alpha + z^2\sin^2\alpha + y^2\sin^2\alpha + 2yz\sin\alpha\cos\alpha + z^2\cos^2\alpha\right)\rho dV \\ &= \int \left(y^2\left(\cos^2\alpha + \sin^2\alpha\right) + z^2\left(\sin^2\alpha + \cos^2\alpha\right)\right)\rho dV \\ &= \int \left(y^2 + z^2\right)\rho dV \\ &= I_x \end{aligned}$$

(17)

Eq 17 shows that when rotating axis *x*, MI relative to axis *x* is invariable.

Substitute Eq 16(a), 16(b) and 16(c) into the sum of MI relative to axis *y* and *z*, i.e. $I_y^\alpha + I_z^\alpha = \int \left(x_\alpha^2 + z_\alpha^2\right)\rho dV + \int \left(x_\alpha^2 + y_\alpha^2\right)\rho dV$, and we will get:

$$I_y^\alpha + I_z^\alpha = \int x^2\rho dV + \int \left(z_\alpha^2 + y_\alpha^2\right)\rho dV + \int x^2\rho dV \quad (18)$$

By Eq 17, Eq 18 can be expressed as:

$$\begin{aligned} I_y^\alpha + I_z^\alpha &= \int x^2 \rho dV + \int \left(z^2 + y^2\right)\rho dV + \int x^2 \rho dV \\ &= \int \left(x^2 + z^2\right)\rho dV + \int \left(x^2 + y^2\right)\rho dV \qquad (19) \\ &= I_y + I_z \end{aligned}$$

Eq 19 shows that by rotating axis x, the MI relative to axis y and z is invariable. Together with Eq 17, by rotating axis x, the MI of the reconstructed CSI is also invariable.

Substitute Eq 16(a) and 16(c) into $I_y^\alpha = \int \left(x_\alpha^2 + z_\alpha^2\right)\rho dV$, and we will get:

$$\begin{aligned} I_y^\alpha &= \int \left(x^2 + (y\sin\alpha + z\cos\alpha)^2\right)\rho dV \\ &= \int \left(x^2 + y^2\sin^2\alpha + 2yz\sin\alpha\cos\alpha + z^2\cos^2\alpha\right)\rho dV \end{aligned} \qquad (20)$$

Substitute Eq 16(a) and 16(b) into $I_z^\alpha = \int \left(x_\alpha^2 + y_\alpha^2\right)\rho dV$, and we will get:

$$\begin{aligned} I_z^\alpha &= \int \left(x^2 + (y\cos\alpha - z\sin\alpha)^2\right)\rho dV \\ &= \int \left(x^2 + y^2\cos^2\alpha - 2yz\sin\alpha\cos\alpha + z^2\sin^2\alpha\right)\rho dV \end{aligned} \qquad (21)$$

Set up the following equation:

$$f(\alpha,\beta,\gamma)_\alpha = I_y^\alpha - I_z^\alpha \qquad (22)$$

By Eq 20 and 21, Eq 22 can be expressed as:

$$f(\alpha,\beta,\gamma)_\alpha = \int \left(y^2\left(\sin^2\alpha - \cos^2\alpha\right) + 4yz\sin\alpha\cos\alpha + z^2\left(\cos^2\alpha - \sin^2\alpha\right)\right)\rho dV$$

(23)

Since $2\sin\alpha\cos\alpha = \sin 2\alpha$, $\cos^2\alpha - \sin^2\alpha = \cos 2\alpha$, Eq 23 can be expressed as:

$$f(\alpha,\beta,\gamma)_\alpha = \int \left(-y^2\cos 2\alpha + 2yz\sin 2\alpha + z^2\cos 2\alpha\right)\rho dV \quad (24)$$

Let

$$\frac{\partial f(\alpha,\beta,\gamma)_\alpha}{\partial \alpha} = 0$$

Change Eq 24 to

$$\begin{aligned} \frac{\partial f(\alpha,\beta,\gamma)_\alpha}{\partial \alpha} &= \frac{\partial\left(\int \left(-y^2\cos 2\alpha + 2yz\sin 2\alpha + z^2\cos 2\alpha\right)\rho dV\right)}{\partial \alpha} \\ &= \int \left(2y^2\sin 2\alpha + 4yz\cos 2\alpha - 2z^2\sin 2\alpha\right)\rho dV = 0 \end{aligned} \qquad (25)$$

Hence,

$$\sin 2\alpha \int y^2 \rho dV + 2\cos 2\alpha \int yz\rho dV - \sin 2\alpha \int z^2 \rho dV = 0 \qquad (26)$$

Since

$$\sin 2\alpha \int x^2 \rho dV - \sin 2\alpha \int x^2 \rho dV = 0 \qquad (27)$$

Substitute Eq 27 into Eq 26, and we will get:

$$\sin 2\alpha \int y^2 \rho dV + \sin 2\alpha \int x^2 \rho dV + 2\cos 2\alpha \int yz\rho dV - \sin 2\alpha \int z^2 \rho dV - \sin 2\alpha \int x^2 \rho dV = 0$$

(28)

By Eq 14, Eq 28 can be expressed as:

$$\sin 2\alpha I_z + 2\cos 2\alpha I_{yz} - \sin 2\alpha I_y = 0 \quad (29)$$

Divide both sides of Eq 27 by $\cos 2\alpha$, and we will get:

$$\tan 2\alpha I_z + 2I_{yz} - \tan 2\alpha I_y = 0 \quad (30)$$

Next, we will get:

$$\tan 2\alpha = \frac{2I_{yz}}{I_y - I_z} \quad (31)$$

Then, get the inverse function of Eq 31:

$$\alpha = \frac{1}{2}\arctan\left(\frac{2I_{yz}}{I_y - I_z}\right) \quad (32)$$

2.2 To rotate around axis *y*

After rotating around axis *x* at α, $ox_\alpha y_\alpha z_\alpha$ are used to stand for the spatial rectangular coordinate system of the reconstructed CSI's COM, which is consisted of finite elements of volume. $I_{xx}^\alpha, I_{yy}^\alpha, I_{zz}^\alpha$ stand for the MI of axis $x_\alpha, y_\alpha, z_\alpha$ respectively.

Their MI will be:

$$\begin{cases} I_{xx}^\alpha = \int (y_\alpha^2 + z_\alpha^2)\rho dV \\ I_{yy}^\alpha = \int (x_\alpha^2 + z_\alpha^2)\rho dV \\ I_{zz}^\alpha = \int (x_\alpha^2 + y_\alpha^2)\rho dV \end{cases} \quad (33)$$

Their PI will be:

$$\begin{cases} I_{xy}^\alpha = \int x_\alpha y_\alpha \rho dV \\ I_{yz}^\alpha = \int y_\alpha z_\alpha \rho dV \\ I_{xz}^\alpha = \int x_\alpha z_\alpha \rho dV \end{cases} \quad (34)$$

where dV stands for element of volume, ρ for the volume's grey value, and $(x_\alpha, y_\alpha, z_\alpha)$ for the position coordinate of the element of volume.

Let the body coordinate system of the reconstructed CSI's COM rotate β around *y*. Then a new coordinate system of $ox_{\alpha\beta} y_{\alpha\beta} z_{\alpha\beta}$ will be formed. The relation between body element of

volume coordinate system of $\left(x_{\alpha\beta}, y_{\alpha\beta}, z_{\alpha\beta}\right)$ and $\left(x_{\alpha}, y_{\alpha}, z_{\alpha}\right)$ will be:

$$\begin{cases} x_{\alpha\beta} = x_{\alpha}\cos\beta + z_{\alpha}\sin\beta & (a) \\ y_{\alpha\beta} = y_{\alpha} & (b) \\ z_{\alpha\beta} = -x_{\alpha}\sin\beta + z_{\alpha}\cos\beta & (c) \end{cases} \qquad (35)$$

Substitute Eq 35(a) and 35(c) into the MI of $I_{yy}^{\alpha\beta} = \int\left(x_{\alpha\beta}^2 + z_{\alpha\beta}^2\right)\rho dV$ relative to axis *y*, and we will get:

$$\begin{aligned} I_{yy}^{\alpha\beta} &= \int\left(\left(x_{\alpha}\cos\beta + z_{\alpha}\sin\beta\right)^2 + \left(-x_{\alpha}\sin\beta + z_{\alpha}\cos\beta\right)^2\right)\rho dV \\ &= \int\left(x_{\alpha}^2\cos^2\beta + 2x_{\alpha}z_{\alpha}\cos\beta\sin\beta + z_{\alpha}^2\sin^2\beta + x_{\alpha}^2\sin^2\beta - 2x_{\alpha}z_{\alpha}\sin\beta\cos\beta + z_{\alpha}^2\cos^2\beta\right)\rho dVm \\ &= \int\left(x_{\alpha}^2\left(\cos^2\beta + \sin^2\beta\right) + z_{\alpha}^2\left(\sin^2\beta + \cos^2\beta\right)\right)\rho dV \\ &= \int\left(x_{\alpha}^2 + z_{\alpha}^2\right)\rho dV \\ &= I_{yy}^{\alpha} \end{aligned}$$

(36)

Eq 36 shows that when rotating axis *y*, the MI relative to axis *y* is invariable.

Substitute Eq 35(a), 35(b) and 35(c) into the sum of the MI relative to axis *x* and *z*, i.e. $I_{xx}^{\alpha\beta} + I_{zz}^{\alpha\beta} = \int\left(y_{\alpha\beta}^2 + z_{\alpha\beta}^2\right)\rho dV + \int\left(x_{\alpha\beta}^2 + y_{\alpha\beta}^2\right)\rho dV$, and we will get:

$$I_{xx}^{\alpha\beta} + I_{zz}^{\alpha\beta} = \int y_{\alpha}^2\rho dV + \int\left(x_{\alpha\beta}^2 + z_{\alpha\beta}^2\right)\rho dV + \int y_{\alpha}^2\rho dV \qquad (37)$$

By Eq 36, Eq 37 can be expressed as:

$$\begin{aligned} I_{xx}^{\alpha\beta} + I_{zz}^{\alpha\beta} &= \int y_{\alpha}^2\rho dV + \int\left(x_{\alpha}^2 + z_{\alpha}^2\right)\rho dV + \int y_{\alpha}^2\rho dV \\ &= \int\left(y_{\alpha}^2 + z_{\alpha}^2\right)\rho dV + \int\left(x_{\alpha}^2 + z_{\alpha}^2\right)\rho dV \qquad (38) \\ &= I_{xx}^{\alpha} + I_{zz}^{\alpha} \end{aligned}$$

Eq 38 shows that by rotating around axis *y*, the MI relative to axis *x* and *z* is invariable. Together with Eq 36, by rotating axis *y*, the MI of the reconstructed CSI is invariable.

Substitute Eq 35(b) and 35(c) into $I_{xx}^{\alpha\beta} = \int\left(y_{\alpha\beta}^2 + z_{\alpha\beta}^2\right)\rho dV$, and we will get:

$$\begin{aligned} I_{xx}^{\alpha\beta} &= \int\left(y_{\alpha\beta}^2 + \left(-x_{\alpha}\sin\beta + z_{\alpha}\cos\beta\right)^2\right)\rho dV \\ &= \int\left(y_{\alpha}^2 + x_{\alpha}^2\sin^2\beta - 2x_{\alpha}z_{\alpha}\sin\beta\cos\beta + z_{\alpha}^2\cos^2\beta\right)\rho dV \end{aligned} \qquad (39)$$

Substitute Eq 35(a) and 35(b) into $I_{zz}^{\alpha\beta} = \int\left(x_{\alpha\beta}^2 + y_{\alpha\beta}^2\right)\rho dV$, and we will get:

$$\begin{aligned} I_{zz}^{\alpha\beta} &= \int\left(\left(x_{\alpha}\cos\beta + z_{\alpha}\sin\beta\right)^2 + y_{\alpha}^2\right)\rho dV \\ &= \int\left(x_{\alpha}^2\cos^2\beta + 2x_{\alpha}z_{\alpha}\sin\beta\cos\beta + z_{\alpha}^2\sin^2\beta + y_{\alpha}^2\right)\rho dV \end{aligned} \qquad (40)$$

Set up the following equation:

$$f\left(\alpha, \beta, \gamma\right)_{\beta} = I_{zz}^{\alpha\beta} - I_{xx}^{\alpha\beta} \qquad (41)$$

By Eq 39 and 40, Eq 41 can be expressed as:

$$f(\alpha,\beta,\gamma)_\beta = \int \left(x_\alpha^2\left(\cos^2\beta - \sin^2\beta\right) + 4x_\alpha z_\alpha \sin\beta\cos\beta - z_\alpha^2\left(\cos^2\beta - \sin^2\beta\right)\right)\rho dV$$

(42)

Since $2\sin\beta\cos\beta = \sin 2\beta$, $\cos^2\beta - \sin^2\beta = \cos 2\beta$, Eq 42 can be expressed as:

$$f(\alpha,\beta,\gamma)_\beta = \int \left(x_\alpha^2 \cos 2\beta + 2x_\alpha z_\alpha \sin 2\beta - z_\alpha^2 \cos 2\beta\right)\rho dV \qquad (43)$$

Let

$$\frac{\partial f(\alpha,\beta,\gamma)_\beta}{\partial \beta} = 0$$

Since

$$\frac{\partial f(\alpha,\beta,\gamma)_\beta}{\partial \beta} = \frac{\partial\left(\int \left(x_\alpha^2 \cos 2\beta + 2x_\alpha z_\alpha \sin 2\beta - z_\alpha^2 \cos 2\beta\right)\rho dV\right)}{\partial \beta} \qquad (44)$$
$$= \int \left(-2x_\alpha^2 \sin 2\beta + 4x_\alpha z_\alpha \cos 2\beta + 2z_\alpha^2 \sin 2\beta\right)\rho dV$$

Hence

$$-\sin 2\beta \int x_\alpha^2 \rho dV + 2\cos 2\beta \int x_\alpha z_\alpha \rho dV + \sin 2\beta \int z_\alpha^2 \rho dV = 0 \qquad (45)$$

Since

$$\sin 2\beta \int y_\alpha^2 \rho dV - \sin 2\beta \int y_\alpha^2 \rho dV = 0 \qquad (46)$$

Substitute Eq 46 into Eq 45, and we will get:

$$-\sin 2\beta \int x_\alpha^2 \rho dV - \sin 2\beta \int y_\alpha^2 \rho dV + 2\cos 2\beta \int x_\alpha z_\alpha \rho dV + \sin 2\alpha \int z_\alpha^2 \rho dV + \sin 2\alpha \int y_\alpha^2 \rho dV = 0$$

(47)

By Eq 33, Eq 47 can be expressed as:

$$-\sin 2\beta I_{zz}^\alpha + 2\cos 2\beta I_{xz}^\alpha + \sin 2\beta I_{xx}^\alpha = 0 \qquad (48)$$

Divide both sides of Eq 48 by $\cos 2\beta$, and we will get:

$$-\tan 2\beta I_{zz}^\alpha + 2\beta I_{xz}^\alpha + \tan 2\beta I_{xx}^\alpha = 0 \qquad (49)$$

Next, we will get:

$$\tan 2\beta = -\frac{2I_{xz}^\alpha}{I_{xx}^\alpha - I_{zz}^\alpha} \qquad (50)$$

Then, get the inverse function of Eq 45:

$$\beta = -\frac{1}{2}\arctan\left(\frac{2I_{xz}^\alpha}{I_{xx}^\alpha - I_{zz}^\alpha}\right) \qquad (51)$$

2.3 To rotate around axis *z*

After rotating around axis *x* at α and then around axis *y* at β, $ox_{\alpha\beta}y_{\alpha\beta}z_{\alpha\beta}$ are used to stand for the spatial rectangular coordinate system of the reconstructed CSI's COM, which is consisted of finite elements of volume. $I_{xx}^{\alpha\beta}, I_{yy}^{\alpha\beta}, I_{zz}^{\alpha\beta}$ stand for the MI of axis $x_{\alpha\beta}, y_{\alpha\beta}, z_{\alpha\beta}$ respectively.

Their MI will be:

$$\begin{cases} I_{xx}^{\alpha\beta} = \int \left(y_{\alpha\beta}^2 + z_{\alpha\beta}^2\right)\rho dV \\ I_{yy}^{\alpha\beta} = \int \left(x_{\alpha\beta}^2 + z_{\alpha\beta}^2\right)\rho dV \\ I_{zz}^{\alpha\beta} = \int \left(x_{\alpha\beta}^2 + y_{\alpha\beta}^2\right)\rho dV \end{cases} \quad (52)$$

Their PI will be:

$$\begin{cases} I_{xy}^{\alpha\beta} = \int x_{\alpha\beta}y_{\alpha\beta}\rho dV \\ I_{yz}^{\alpha\beta} = \int y_{\alpha\beta}z_{\alpha\beta}\rho dV \\ I_{xz}^{\alpha\beta} = \int x_{\alpha\beta}z_{\alpha\beta}\rho dV \end{cases} \quad (53)$$

where dV stands for element of volume, ρ for the volume's grey value, and $\left(x_{\alpha\beta}, y_{\alpha\beta}, z_{\alpha\beta}\right)$ for the position coordinate of the element of volume.

Let the body coordinate system of the reconstructed CSI's COM rotate γ around *z*. Then a new coordinate system of $ox_{\alpha\beta\gamma}y_{\alpha\beta\gamma}z_{\alpha\beta\gamma}$ will be formed. The relation between body element of volume coordinate system of $\left(x_{\alpha\beta\gamma}, y_{\alpha\beta\gamma}, z_{\alpha\beta\gamma}\right)$ and $\left(x_{\alpha\beta}, y_{\alpha\beta}, z_{\alpha\beta}\right)$ will be:

Rotate γ around axis *z*, the relation between the old and new coordinate systems will be:

$$\begin{cases} x_{\alpha\beta\gamma} = x_{\alpha\beta}\cos\gamma - y_{\alpha\beta}\sin\gamma & (a) \\ y_{\alpha\beta\gamma} = x_{\alpha\beta}\sin\gamma + y_{\alpha\beta}\cos\gamma & (b) \\ z_{\alpha\beta\gamma} = z_{\alpha\beta} & (c) \end{cases} \quad (54)$$

Substitute Eq 54(a) and 54(b) into the MI of $I_{zz}^{\alpha\beta\gamma} = \int \left(x_{\alpha\beta\gamma}^2 + y_{\alpha\beta\gamma}^2\right)\rho dV$ relative to axis *z*, and we will get:

$$\begin{aligned} I_{zz}^{\alpha\beta\gamma} &= \int \left(\left(x_{\alpha\beta}\cos\gamma - y_{\alpha\beta}\sin\gamma\right)^2 + \left(x_{\alpha\beta}\sin\gamma + y_{\alpha\beta}\cos\gamma\right)^2\right)\rho dV \\ &= \int \left(x_{\alpha\beta}^2\cos^2\gamma - 2x_{\alpha\beta}y_{\alpha\beta}\cos\gamma\sin\gamma + y_{\alpha\beta}^2\sin^2\gamma + x_{\alpha\beta}^2\sin^2\gamma + 2x_{\alpha\beta}y_{\alpha\beta}\sin\gamma\cos\gamma + y_{\alpha\beta}^2\cos^2\gamma\right)\rho dV \\ &= \int \left(x_{\alpha\beta}^2\left(\cos^2\gamma + \sin^2\gamma\right) + y_{\alpha\beta}^2\left(\sin^2\gamma + \cos^2\gamma\right)\right)\rho dV \\ &= \int \left(x_{\alpha\beta}^2 + y_{\alpha\beta}^2\right)\rho dV \\ &= I_{zz}^{\alpha\beta} \end{aligned}$$

(55)

Eq 55 shows that when rotating around axis *z*, the PI relative to axis *z* is invariable.

Substitute Eq 54(a), 54(b) and 54(c) into the sum of PI, i.e. $I_{xx}^{\alpha\beta\gamma} + I_{yy}^{\alpha\beta\gamma} = \int\left(y_{\alpha\beta\gamma}^2 + z_{\alpha\beta\gamma}^2\right)\rho dV + \int\left(x_{\alpha\beta\gamma}^2 + z_{\alpha\beta\gamma}^2\right)\rho dV$ relative to axis *x* and axis *z*, and we will get:

$$\begin{aligned}
I_{xx}^{\alpha\beta\gamma} + I_{yy}^{\alpha\beta\gamma} &= \int z_{\alpha\beta}^2 \rho dV + \int\left(x_{\alpha\beta\gamma}^2 + y_{\alpha\beta\gamma}^2\right)\rho dV + \int z_{\alpha\beta}^2 \rho dV \\
&= \int z_{\alpha\beta}^2 \rho dV + \int\left(x_{\alpha\beta}^2 + y_{\alpha\beta}^2\right)\rho dV + \int z_{\alpha\beta}^2 \rho dV \\
&= \int\left(y_{\alpha\beta}^2 + z_{\alpha\beta}^2\right)\rho dV + \int\left(x_{\alpha\beta}^2 + z_{\alpha\beta}^2\right)\rho dV \\
&= I_{xx}^{\alpha\beta} + I_{yy}^{\alpha\beta}
\end{aligned} \qquad (56)$$

Eq 56 shows that when rotating around axis *z*, the PI relative to axis *x* and axis *y* is invariable. Together with Eq 55, by rotating axis *z*, the MI of the reconstructed CSI is invariable.

Substitute Eq 54(b) and 54(c) into $I_{xx}^{\alpha\beta\gamma} = \int\left(y_{\alpha\beta\gamma}^2 + z_{\alpha\beta\gamma}^2\right)\rho dV$, and we will get:

$$\begin{aligned}
I_{xx}^{\alpha\beta\gamma} &= \int\left(\left(x_{\alpha\beta}\sin\gamma + y_{\alpha\beta}\cos\gamma\right)^2 + z_{\alpha\beta}^2\right)\rho dV \\
&= \int\left(x_{\alpha\beta}^2\sin^2\gamma + 2x_{\alpha\beta}y_{\alpha\beta}\sin\gamma\cos\gamma + y_{\alpha\beta}^2\cos^2\gamma + z_{\alpha\beta}^2\right)\rho dV
\end{aligned} \qquad (57)$$

Substitute Eq 54(a) and 54(b) into $I_{yy}^{\alpha\beta\gamma} = \int\left(x_{\alpha\beta\gamma}^2 + z_{\alpha\beta\gamma}^2\right)\rho dV$, and we will get:

$$\begin{aligned}
I_{yy}^{\alpha\beta\gamma} &= \int\left(\left(x_{\alpha\beta}\cos\gamma - y_{\alpha\beta}\sin\gamma\right)^2 + z_{\alpha\beta}^2\right)\rho dV \\
&= \int\left(x_{\alpha\beta}^2\cos^2\gamma - 2x_{\alpha\beta}y_{\alpha\beta}\sin\gamma\cos\gamma + y_{\alpha\beta}^2\sin^2\gamma + z_{\alpha\beta}^2\right)\rho dV
\end{aligned} \qquad (58)$$

Set up the following equation:

$$f(\alpha,\beta,\gamma)_\gamma = I_{xx}^{\alpha\beta\gamma} - I_{yy}^{\alpha\beta\gamma} \qquad (59)$$

By Eq 57 and 58, Eq 59 can be expressed as:

$$f(\alpha,\beta,\gamma)_\gamma = \int \left(-x_{\alpha\beta}^2\left(\cos^2\gamma - \sin^2\gamma\right) + 4x_{\alpha\beta}y_{\alpha\beta}\sin\gamma\cos\gamma + y_{\alpha\beta}^2\left(\cos^2\gamma - \sin^2\gamma\right)\right)\rho dV$$

(60)

Since $2\sin\gamma\cos\lambda = \sin 2\gamma$, $\cos^2\gamma - \sin^2\gamma = \cos 2\gamma$, Eq 60 can be expressed as:

$$f(\alpha,\beta,\gamma)_\gamma = \int \left(-x_{\alpha\beta}^2\cos 2\gamma + 2x_{\alpha\beta}y_{\alpha\beta}\sin 2\gamma + y_{\alpha\beta}^2\cos 2\gamma\right)\rho dV \qquad (61)$$

Let

$$\frac{\partial f(\alpha,\beta,\gamma)_\gamma}{\partial\gamma} = 0$$

Since

$$\frac{\partial f(\alpha,\beta,\gamma)_\gamma}{\partial\gamma} = \frac{\partial\left(\int\left(-x_{\alpha\beta}^2\cos 2\gamma + 2x_{\alpha\beta}y_{\alpha\beta}\sin 2\gamma + y_{\alpha\beta}^2\cos 2\gamma\right)\rho dV\right)}{\partial\gamma} \qquad (62)$$
$$= \int\left(2x_{\alpha\beta}^2\sin 2\gamma + 4x_{\alpha\beta}y_{\alpha\beta}\cos 2\gamma - 2y_{\alpha\beta}^2\cos 2\gamma\right)\rho dV$$

Hence

$$\sin 2\gamma\int x_{\alpha\beta}^2\rho dV + 2\cos 2\gamma\int x_{\alpha\beta}y_{\alpha\beta}\rho dV - \sin 2\gamma\int y_{\alpha\beta}^2\rho dV = 0 \qquad (63)$$

Since

$$\sin 2\gamma\int z_{\alpha\beta}^2\rho dV - \sin 2\gamma\int z_{\alpha\beta}^2\rho dV = 0 \qquad (64)$$

Substitute Eq 64 into Eq 63, we will get:

$$\sin 2\gamma\int x_{\alpha\beta}^2\rho dV + \sin 2\gamma\int z_{\alpha\beta}^2\rho dV + 2\cos 2\gamma\int x_{\alpha\beta}y_{\alpha\beta}\rho dV - \sin 2\gamma\int y_{\alpha\beta}^2\rho dV - \sin 2\gamma\int z_{\alpha\beta}^2\rho dV = 0$$

(65)

By Eq 52, Eq 65 can be expressed as:

$$\sin 2\gamma I_{yy}^{\alpha\beta} + 2\cos 2\gamma I_{xy}^{\alpha\beta} - \sin 2\gamma I_{xx}^{\alpha\beta} = 0 \qquad (66)$$

Divide both sides of Eq 66 by $\cos 2\gamma$, and we will get:

$$\tan 2\gamma I_{yy}^{\alpha\beta} + 2I_{xy}^{\alpha\beta} - \tan 2\gamma I_{xx}^{\alpha\beta} = 0 \qquad (67)$$

Next, we will get:

$$\tan 2\gamma = \frac{2I_{xy}^{\alpha\beta}}{I_{xx}^{\alpha\beta} - I_{yy}^{\alpha\beta}} \qquad (68)$$

Then, get the inverse function of Eq 68:

$$\gamma = \frac{1}{2}\arctan\left(\frac{2I_{xy}^{\alpha\beta}}{I_{xx}^{\alpha\beta} - I_{yy}^{\alpha\beta}}\right) \qquad (69)$$

3 The order of rotation of the reconstructed CSI and its PAI and MI

3.1 Rotation of $x \to y \to z$

By Eq 16, Eq 35 can be expressed as:

$$\begin{cases} x_{\alpha\beta} = x\cos\beta + (y\sin\alpha + z\cos\alpha)\sin\beta & (a) \\ y_{\alpha\beta} = y\cos\alpha - z\sin\alpha & (b) \\ z_{\alpha\beta} = -x\sin\beta + (y\sin\alpha + z\cos\alpha)\cos\beta & (c) \end{cases} \quad (70)$$

By Eq 54, Eq 70 can be expressed as:

$$\begin{cases} x_{\alpha\beta\gamma} = (x\cos\beta + y\sin\alpha\sin\beta + z\cos\alpha\sin\beta)\cos\gamma - (y\cos\alpha - z\sin\alpha)\sin\gamma & (a) \\ y_{\alpha\beta\gamma} = (x\cos\beta + y\sin\alpha\sin\beta + z\cos\alpha\sin\beta)\sin\gamma + (y\cos\alpha - z\sin\alpha)\cos\gamma & (b) \\ z_{\alpha\beta\gamma} = -x\sin\beta + y\sin\alpha\cos\beta + z\cos\alpha\cos\beta & (c) \end{cases}$$

(71)

3.2 Rotation of $y \to z \to x$

By Eq 54, Eq 35 can be expressed as:

$$\begin{cases} x_{\beta\gamma} = (x\cos\beta + z\sin\beta)\cos\gamma - y\sin\gamma & (a) \\ y_{\beta\gamma} = (x\cos\beta + z\sin\beta)\sin\gamma + y\cos\gamma & (b) \\ z_{\beta\gamma} = -x\sin\beta + z\cos\beta & (c) \end{cases} \quad (72)$$

By Eq 16, Eq 72 can be expressed as:

$$\begin{cases} x_{\beta\gamma\alpha} = (x\cos\beta + z\sin\beta)\cos\gamma - y\sin\gamma & (a) \\ y_{\beta\gamma\alpha} = (x\cos\beta\sin\gamma + z\sin\beta\sin\gamma + y\cos\gamma)\cos\alpha - (z\cos\beta - x\sin\beta)\sin\alpha & (b) \\ z_{\beta\gamma\alpha} = (x\cos\beta\sin\gamma + z\sin\beta\sin\gamma + y\cos\gamma)\sin\alpha + (z\cos\beta - x\sin\beta)\cos\alpha & (c) \end{cases}$$

(73)

3.3 Rotation of $z \to x \to y$

By Eq 16, Eq 54 can be expressed as:

$$\begin{cases} x_{\gamma\alpha} = x\cos\gamma - y\sin\gamma & (a) \\ y_{\gamma\alpha} = (x\sin\gamma + y\cos\gamma)\cos\alpha - z\sin\alpha & (b) \\ z_{\gamma\alpha} = (x\sin\gamma + y\cos\gamma)\sin\alpha + z\cos\alpha & (c) \end{cases} \quad (74)$$

By Eq 35, Eq 74 can be expressed as:

$$\begin{cases} x_{\gamma\alpha\beta} = (x\cos\gamma - y\sin\gamma)\cos\beta + (x\sin\gamma\sin\alpha + y\cos\gamma\sin\alpha + z\cos\alpha)\sin\beta & (a) \\ y_{\gamma\alpha\beta} = (x\sin\gamma\cos\alpha + y\cos\gamma\cos\alpha - z\sin\alpha & (b) \\ z_{\gamma\alpha\beta} = -(x\cos\gamma - y\sin\gamma)\sin\beta + (x\sin\gamma\sin\alpha + y\cos\gamma\sin\alpha + z\cos\alpha)\cos\beta & (c) \end{cases}$$

(75)

Eq 71, 73 and 75 show that with different rotation order, the results of the positioning of bone *in vivo* differ.
