# Supplementary material for: Study of Sexual Dimorphism in Metatarsal Bones: Geometric and Inertial Analysis of the Three-Dimensional Reconstructed Models
Source: Front Endocrinol (Lausanne). 2021 Oct 14;12:734362. doi: 10.3389/fendo.2021.734362 (PMC8551807; doi:10.3389/fendo.2021.734362)
Supplement: Supplementary file 3 [file DataSheet_3.pdf]

Supplementary File III

Table S1 Characteristics of subjects

Characteristics	Males (N=30)			Females (N=30)			Total (N=60)		
	**Mean**	**SD**	**Median**	**Mean**	**SD**	**Median**	**Mean**	**SD**	**Median**
**Age (years)**	21.8	3.6	20.5	20.0	1.9	19.0	20.9	3.0	20.0
**Body height (cm)**	176.6	10.0	175.5	165.2	5.9	165.5	170.9	9.9	169.5
**Body weight (kg)**	69.8	9.1	68.7	55.2	5.8	55.5	62.5	10.6	61.0

Table S2 Intraclass correlation coefficient of metatarsal bones between two scans

Metatarsals	Length	Width	Height	Volume	Surface Area	SA: V
**1st**	1.00	0.98	0.99	0.99	0.99	0.97
**2nd**	1.00	0.98	0.97	0.98	0.98	0.98
**3rd**	1.00	0.91	0.93	0.81	0.91	0.93
**4th**	1.00	0.97	0.97	0.95	0.96	0.96
**5th**	1.00	0.91	0.99	0.97	0.99	0.97

(Data from Liu et al., 2018)

Reference:

Liu, Y., Li, R., Fan, Y., Antonijević, Đ., Milenković, P., Li, Z., et al. (2018). The influence of anisotropic voxel caused by field of view setting on the accuracy of three-dimensional reconstruction of bone geometric models. *AIP Adv*. 8, 085111. doi: 10.1063/1.50419

Table S3 Normality and homoscedasticity tests of normalized length, width and height of metatarsal bone *in vivo* based on its PAI between sexes

Metatarsals	Geometric parameters		Shapiro-Wilk test				Levene's Test	
			Statistics (male)	Sig (male)	Statistics (female)	Sig (female)	F	Sig
1st	Length	L	0.963	0.365	0.982	0.865	2.078	0.155
		R	0.973	0.626	0.977	0.732	0.816	0.370
	Width	L	0.946	0.130	0.922	0.030*	3.099	0.084
		R	0.894	0.006**	0.974	0.647	1.970	0.166
	Height	L	0.986	0.956	0.980	0.816	1.175	0.283
		R	0.983	0.905	0.953	0.199	0.116	0.735
2nd	Length	L	0.978	0.763	0.972	0.604	4.413	0.040*
		R	0.973	0.636	0.940	0.093	2.150	0.148
	Width	L	0.971	0.569	0.969	0.504	3.387	0.071
		R	0.985	0.939	0.953	0.207	0.933	0.338
	Height	L	0.974	0.650	0.902	0.009**	2.380	0.128
		R	0.989	0.985	0.977	0.746	0.633	0.430
3rd	Length	L	0.971	0.564	0.972	0.605	0.924	0.340
		R	0.965	0.406	0.946	0.135	1.505	0.225
	Width	L	0.976	0.699	0.947	0.139	0.722	0.399
		R	0.953	0.207	0.984	0.912	0.439	0.510
	Height	L	0.958	0.283	0.935	0.068	0.024	0.876
		R	0.970	0.547	0.982	0.866	0.078	0.781
4th	Length	L	0.986	0.956	0.979	0.789	0.692	0.409
		R	0.987	0.966	0.977	0.742	1.527	0.222
	Width	L	0.984	0.918	0.913	0.018*	0.068	0.795
		R	0.954	0.213	0.950	0.166	0.252	0.618
	Height	L	0.976	0.700	0.982	0.873	0.479	0.492
		R	0.965	0.404	0.975	0.693	2.254	0.139
5th	Length	L	0.905	0.011	0.970	0.552	0.421	0.519
		R	0.950	0.165	0.980	0.834	0.712	0.402
	Width	L	0.949	0.159	0.987	0.968	2.163	0.147
		R	0.916	0.022*	0.974	0.665	4.340	0.042*
	Height	L	0.964	0.384	0.983	0.905	0.006	0.939
		R	0.936	0.069	0.969	0.506	0.030	0.862

* Significance level: $p<0.05$; ** Significance level: $p<0.01$

Table S4 Normality and homoscedasticity tests of SA: V (mm^{-1}) and bone density (HU/1024) of metatarsal bone in vivo between sexes

Metatarsals	Geometric parameters		Shapiro-Wilk test				Levene's Test	
			Statistics (male)	Sig (male)	Statistics (female)	Sig (female)	F	Sig
1st	SA: V	L	0.962	0.351	0.979	0.796	0.510	0.478
		R	0.981	0.849	0.981	0.860	0.350	0.557
	Density	L	0.967	0.458	0.979	0.812	0.628	0.431
		R	0.983	0.903	0.968	0.480	1.631	0.207
2nd	SA: V	L	0.946	0.134	0.973	0.613	0.576	0.451
		R	0.941	0.096	0.975	0.673	2.372	0.129
	Density	L	0.985	0.932	0.991	0.993	0.094	0.761
		R	0.991	0.993	0.975	0.676	0.677	0.414
3rd	SA: V	L	0.975	0.687	0.976	0.721	0.143	0.707
		R	0.961	0.321	0.979	0.792	1.205	0.277
	Density	L	0.989	0.986	0.964	0.392	0.175	0.678
		R	0.986	0.959	0.976	0.714	0.369	0.546
4th	SA: V	L	0.971	0.572	0.972	0.600	2.747	0.103
		R	0.963	0.377	0.978	0.765	4.271	0.043*
	Density	L	0.952	0.197	0.971	0.576	1.156	0.287
		R	0.898	0.007**	0.977	0.730	0.698	0.407
5th	SA: V	L	0.982	0.878	0.975	0.691	1.614	0.209
		R	0.960	0.319	0.956	0.246	1.221	0.274
	Density	L	0.932	0.056	0.969	0.510	1.913	0.172
		R	0.920	0.026*	0.970	0.541	1.887	0.175

* Significance level: $p<0.05$; ** Significance level: $p<0.01$

Table S5 Normality and homoscedasticity tests of normalized PMIs of metatarsal bone in vivo based on its PAI between sexes

Metatarsals	Geometric parameters		Shapiro-Wilk test				Levene's Test	
			Statistics (male)	Sig (male)	Statistics (female)	Sig (female)	F	Sig
1st	PMI*x*	L	0.939	0.083	0.960	0.309	4.107	0.047*
		R	0.952	0.187	0.962	0.356	11.588	0.001**
	PMI*y*	L	0.937	0.077	0.946	0.129	3.604	0.063
		R	0.962	0.353	0.952	0.192	10.854	0.002**
	PMIz	L	0.947	0.144	0.985	0.937	4.485	0.038*
		R	0.971	0.567	0.967	0.464	8.550	0.005**
2nd	PMI*x*	L	0.957	0.267	0.971	0.559	2.585	0.113
		R	0.988	0.975	0.948	0.147	5.756	0.020*
	PMI*y*	L	0.980	0.836	0.953	0.207	3.654	0.061
		R	0.961	0.329	0.977	0.747	1.991	0.164
	PMIz	L	0.968	0.486	0.971	0.560	3.672	0.060
		R	0.994	1.000	0.954	0.222	4.863	0.031*
3rd	PMI*x*	L	0.953	0.206	0.971	0.572	1.165	0.285
		R	0.970	0.528	0.941	0.099	2.995	0.089
	PMI*y*	L	0.972	0.596	0.988	0.980	12.701	0.001**
		R	0.977	0.746	0.932	0.054	14.182	0.000**
	PMIz	L	0.972	0.593	0.957	0.259	3.515	0.066
		R	0.977	0.734	0.934	0.064	7.814	0.007**
4th	PMI*x*	L	0.976	0.710	0.963	0.372	4.994	0.029*
		R	0.981	0.864	0.970	0.545	3.802	0.056
	PMI*y*	L	0.984	0.926	0.891	0.005**	4.784	0.033*
		R	0.984	0.919	0.834	0.000**	0.083	0.774
	PMIz	L	0.958	0.282	0.938	0.083	6.714	0.012*
		R	0.968	0.483	0.937	0.078	4.364	0.041*
5th	PMI*x*	L	0.967	0.458	0.950	0.172	4.049	0.049*
		R	0.972	0.598	0.956	0.243	5.526	0.022*
	PMI*y*	L	0.946	0.134	0.963	0.365	1.853	0.179
		R	0.983	0.902	0.943	0.112	4.405	0.040*
	PMIz	L	0.946	0.136	0.923	0.033*	4.757	0.033*
		R	0.990	0.993	0.940	0.090	4.821	0.032*

* Significance level: $p<0.05$; ** Significance level: $p<0.01$

Table S6 Correlation results between variables of the 1st metatarsal of the left side

		Length	Width	Height	S: V	Density	PMI*x*	PMI*y*	PMI*z*	Height	Weight	Age
**Length**	**Pearson Coefficient**	1	-0.785**	-0.843**	0.219	-0.074	0.792**	0.623**	-0.817**	0.162	-0.136	-0.007
	**Sig. (2-tailed)**		0.000	0.000	0.0093	0.572	0.000	0.000	0.000	0.217	0.300	0.959
	**N**	60	60	60	60	60	60	60	60	60	60	60
**Width**	**Pearson Coefficient**	-0.785**	1	0.329*	-0.182	0.165	-0.608**	-0.740**	-0.786**	-0.032	0.231	-0.015
	**Sig. (2-tailed)**	0.000		0.01	0.164	0.207	0.000	0.000	0.000	0.807	0.076	0.908
	**N**	60	60	60	60	60	60	60	60	60	60	60
**Height**	**Pearson Coefficient**	-0.843**	0.329*	1	-0.176	-0.030	-0.679**	-0.307*	-0.563**	-0.176	0.067	0.047
	**Sig. (2-tailed)**	0.000	0.01		0.180	0.818	0.000	0.017	0.000	0.179	0.612	0.724
	**N**	60	60	60	60	60	60	60	60	60	60	60
**S: V**	**Pearson Coefficient**	0.219	-0.182	-0.176	1	0.155	0.304*	0.365**	-0.390**	-0.767**	-0.748**	-0.304*
	**Sig. (2-tailed)**	0.0093	0.164	0.180		0.236	0.018	0.004	0.002	0.000	0.000	0.018
	**N**	60	60	60	60	60	60	60	60	60	60	60
**Density**	**Pearson Coefficient**	-0.074	0.165	-0.030	0.155	1	-0.015	-0.129	0.087	-0.026	0.064	-0.082
	**Sig. (2-tailed)**	0.572	0.207	0.818	0.236		0.907	0.327	0.511	0.841	0.629	0.533
	**N**	60	60	60	60	60	60	60	60	60	60	60
**PMI*x***	**Pearson Coefficient**	0.792**	-0.608**	-0.679**	0.304*	-0.015	1	0.483**	-0.847**	0.095	-0.134	-0.069
	**Sig. (2-tailed)**	0.000	0.000	0.000	0.018	0.907		0.000	0.000	0.468	0.307	0.601
	**N**	60	60	60	60	60	60	60	60	60	60	60
**PMI*y***	**Pearson Coefficient**	0.623**	-0.740**	-0.307*	0.365**	-0.129	0.483**	1	-0.874**	-0.157	-0.385**	-0.012
	**Sig. (2-tailed)**	0.000	0.000	0.017	0.004	0.327	0.000		0.000	0.231	0.002	0.926
	**N**	60	60	60	60	60	60	60	60	60	60	60
**PMI*z***	**Pearson Coefficient**	-0.817**	-0.786**	-0.563**	-0.390**	0.087	-0.847**	-0.874**	1	0.039	0.296*	0.129
	**Sig. (2-tailed)**	0.000	0.000	0.000	0.002	0.511	0.000	0.000		0.768	0.022	0.324
	**N**	60	60	60	60	60	60	60	60	60	60	60
**Height**	**Spearman Coefficient**	0.162	-0.032	-0.176	-0.767**	-0.026	0.095	-0.157	0.039	1	0.788**	0.259*
	**Sig. (2-tailed)**	0.217	0.807	0.179	0.000	0.841	0.468	0.231	0.768		0.000	0.045
	**N**	60	60	60	60	60	60	60	60	60	60	60
**Weight**	**Spearman Coefficient**	-0.136	0.231	0.067	-0.748**	0.064	-0.134	-0.385**	0.296*	0.788**	1	0.198
	**Sig. (2-tailed)**	0.300	0.076	0.612	0.000	0.629	0.307	0.002	0.022	0.000		0.130
	**N**	60	60	60	60	60	60	60	60	60	60	60
**Age**	**Spearman Coefficient**	-0.007	-0.015	0.047	-0.304*	-0.082	-0.069	-0.012	0.129	0.259*	0.198	1
	**Sig. (2-tailed)**	0.959	0.908	0.724	0.018	0.533	0.601	0.926	0.324	0.045	0.130	
	**N**	60	60	60	60	60	60	60	60	60	60	60

Table S7 Correlation results between variables of the 2nd metatarsal of the left side

		**Length**	**Width**	**Height**	**S: V**	**Density**	**PMI*x***	**PMI*y***	**PMIz**	**Height**	**Weight**	**Age**
**Length**	**Pearson Coefficient**	1	-0.714**	-0.857**	0.253	-0.055	0.806**	0.736**	-0.838**	0.058	-0.131	-0.054
	**Sig. (2-tailed)**		0.000	0.000	0.051	0.675	0.000	0.000	0.000	0.661	0.317	0.684
	**N**	60	60	60	60	60	60	60	60	60	60	60
**Width**	**Pearson Coefficient**	-0.714**	1	0.252	-0.238	0.018	-0.381**	-0.734**	0.554**	-0.057	0.045	0.095
	**Sig. (2-tailed)**	0.000		0.052	0.067	0.892	0.003	0.000	0.000	0.666	0.734	0.471
	**N**	60	60	60	60	60	60	60	60	60	60	60
**Height**	**Pearson Coefficient**	-0.857**	0.252	1	-0.175	0.063	-0.835**	-0.478**	0.751**	-0.051	0.134	-0.008
	**Sig. (2-tailed)**	0.000	0.052		0.181	0.632	0.000	0.000	0.000	0.698	0.308	0.952
	**N**	60	60	60	60	60	60	60	60	60	60	60
**S: V**	**Pearson Coefficient**	0.253	-0.238	-0.175	1	0.205	0.386**	0.382**	-0.413**	-0.791**	-0.775**	-0.188
	**Sig. (2-tailed)**	0.051	0.067	0.181		0.116	0.002	0.003	0.001	0.000	0.000	0.151
	**N**	60	60	60	60	60	60	60	60	60	60	60
**Density**	**Pearson Coefficient**	-0.055	0.018	0.063	0.205	1	0.067	0.159	-0.109	-0.232	-0.253	-0.201
	**Sig. (2-tailed)**	0.675	0.892	0.632	0.116		0.613	0.226	0.405	0.075	0.051	0.124
	**N**	60	60	60	60	60	60	60	60	60	60	60
**PMI*x***	**Pearson Coefficient**	0.806**	-0.381**	-0.835**	0.386**	0.067	1	0.715**	-0.958**	-0.162	-0.362**	-0.070
	**Sig. (2-tailed)**	0.000	0.003	0.000	0.002	0.613		0.000	0.000	0.215	0.005	0.598
	**N**	60	60	60	60	60	60	60	60	60	60	60
**PMI*y***	**Pearson Coefficient**	0.736**	-0.734**	-0.478**	0.382**	0.159	0.715**	1	-0.886**	-0.084	-0.246	-0.095
	**Sig. (2-tailed)**	0.000	0.000	0.000	0.003	0.226	0.000		0.000	0.521	0.058	0.471
	**N**	60	60	60	60	60	60	60	60	60	60	60
**PMIz**	**Pearson Coefficient**	-0.838**	0.554**	0.751**	-0.413**	-0.109	-0.958**	-0.886**	1	0.137	0.340**	0.071
	**Sig. (2-tailed)**	0.000	0.000	0.000	0.001	0.405	0.000	0.000		0.296	0.008	0.591
	**N**	60	60	60	60	60	60	60	60	60	60	60
**Height**	**Spearman Coefficient**	0.058	-0.057	-0.051	-0.791**	-0.232	-0.162	-0.084	0.137	1	0.788**	0.259*
	**Sig. (2-tailed)**	0.661	0.666	0.698	0.000	0.075	0.215	0.521	0.296		0.000	0.045
	**N**	60	60	60	60	60	60	60	60	60	60	60
**Weight**	**Spearman Coefficient**	-0.131	0.045	0.134	-0.775**	-0.253	-0.362**	-0.246	0.340**	0.788**	1	0.198
	**Sig. (2-tailed)**	0.317	0.734	0.308	0.000	0.051	0.005	0.058	0.008	0		0.130
	**N**	60	60	60	60	60	60	60	60	60	60	60
**Age**	**Spearman Coefficient**	-0.054	0.095	-0.008	-0.188	-0.201	-0.070	-0.095	0.071	0.259*	0.198	1
	**Sig. (2-tailed)**	0.684	0.471	0.952	0.151	0.124	0.598	0.471	0.591	0.045	0.130	
	**N**	60	60	60	60	60	60	60	60	60	60	60

Table S8 Correlation results between variables of the 3rd metatarsal of the left side

		Length	Width	Height	S: V	Density	PMI*x*	PMI*y*	PMIz	Height	Weight	Age
**Length**	**Pearson Coefficient**	1	-0.744**	-0.839**	0.322*	-0.142	0.804**	0.714**	-0.820**	0.140	-0.125	0.029
	**Sig. (2-tailed)**		0.000	0.000	0.012	0.280	0.000	0.000	0.000	0.285	0.341	0.828
	**N**	60	60	60	60	60	60	60	60	60	60	60
**Width**	**Pearson Coefficient**	-0.744**	1	0.260*	-0.271*	0.076	-0.415**	-0.687**	0.550**	-0.051	0.059	0.095
	**Sig. (2-tailed)**	0.000		0.044	0.036	0.562	0.001	0.000	0.000	0.698	0.656	0.469
	**N**	60	60	60	60	60	60	60	60	60	60	60
**Height**	**Pearson Coefficient**	-0.839**	0.260*	1	-0.244	0.143	-0.824**	-0.472**	0.737**	-0.203	0.104	-0.098
	**Sig. (2-tailed)**	0.000	0.044		0.060	0.277	0.000	0.000	0.000	0.120	0.430	0.454
	**N**	60	60	60	60	60	60	60	60	60	60	60
**S: V**	**Pearson Coefficient**	0.322*	-0.271*	-0.244	1	0.105	0.358**	0.423**	-0.407**	-0.654**	-0.801**	-0.245
	**Sig. (2-tailed)**	0.012	0.036	0.060		0.423	0.005	0.001	0.001	0.000	0.000	0.059
	**N**	60	60	60	60	60	60	60	60	60	60	60
**Density**	**Pearson Coefficient**	-0.142	0.076	0.143	0.105	1	-0.020	0.014	0.008	-0.258*	-0.169	-0.027
	**Sig. (2-tailed)**	0.280	0.562	0.277	0.423		0.879	0.917	0.952	0.047	0.198	0.837
	**N**	60	60	60	60	60	60	60	60	60	60	60
**PMI*x***	**Pearson Coefficient**	0.804**	-0.415**	-0.824**	0.358**	-0.020	1	0.750**	-0.965**	0.037	-0.249	-0.001
	**Sig. (2-tailed)**	0.000	0.001	0.000	0.005	0.879		0.000	0.000	0.782	0.055	0.993
	**N**	60	60	60	60	60	60	60	60	60	60	60
**PMI*y***	**Pearson Coefficient**	0.714**	-0.687**	-0.472**	0.423**	0.014	0.750**	1	-0.897**	0.008	-0.213	-0.133
	**Sig. (2-tailed)**	0.000	0.000	0.000	0.001	0.917	0.000		0.000	0.949	0.102	0.309
	**N**	60	60	60	60	60	60	60	60	60	60	60
**PMIz**	**Pearson Coefficient**	-0.820**	0.550**	0.737**	-0.407**	0.008	-0.965**	-0.897**	1	-0.013	0.274*	0.051
	**Sig. (2-tailed)**	0.000	0.000	0.000	0.001	0.952	0.000	0.000		0.921	0.034	0.699
	**N**	60	60	60	60	60	60	60	60	60	60	60
**Height**	**Spearman Coefficient**	0.140	-0.051	-0.203	-0.654**	-0.258*	0.037	0.008	-0.013	1	0.788**	0.259*
	**Sig. (2-tailed)**	0.285	0.698	0.120	0.000	0.047	0.782	0.949	0.921		0.000	0.045
	**N**	60	60	60	60	60	60	60	60	60	60	60
**Weight**	**Spearman Coefficient**	-0.125	0.059	0.104	-0.801**	-0.169	-0.249	-0.213	0.274*	0.788**	1	0.198
	**Sig. (2-tailed)**	0.341	0.656	0.430	0.000	0.198	0.055	0.102	0.034	0.000		0.130
	**N**	60	60	60	60	60	60	60	60	60	60	60
**Age**	**Spearman Coefficient**	0.029	0.095	-0.098	-0.245	-0.027	-0.001	-0.133	0.051	0.259*	0.198	1
	**Sig. (2-tailed)**	0.828	0.469	0.454	0.059	0.837	0.993	0.309	0.699	0.045	0.130	
	**N**	60	60	60	60	60	60	60	60	60	60	60

Table S9 Correlation results between variables of the 4th metatarsal of the left side

		Length	Width	Height	S: V	Density	PMI*x*	PMI*y*	PMI*z*	Height	Weight	Age
**Length**	**Pearson Coefficient**	1	-0.632**	-0.774**	0.424**	-0.009	0.819**	0.740**	-0.846**	0.024	-0.162	-0.104
	**Sig. (2-tailed)**		0.000	0.000	0.001	0.945	0.000	0.000	0.000	0.855	0.215	0.429
	**N**	60	60	60	60	60	60	60	60	60	60	60
**Width**	**Pearson Coefficient**	-0.632**	1	-0.002	-0.233	0.014	-0.280*	-0.613**	0.447**	0.052	0.090	0.276*
	**Sig. (2-tailed)**	0.000		0.989	0.073	0.916	0.030	0.000	0.000	0.693	0.492	0.033
	**N**	60	60	60	60	60	60	60	60	60	60	60
**Height**	**Pearson Coefficient**	-0.774**	-0.002	1	-0.356**	0.000	-0.828**	-0.453**	0.726**	-0.013	0.173	-0.043
	**Sig. (2-tailed)**	0.000	0.989		0.005	0.998	0.000	0.000	0.000	0.922	0.187	0.743
	**N**	60	60	60	60	60	60	60	60	60	60	60
**S: V**	**Pearson Coefficient**	0.424**	-0.233	-0.356**	1	0.056	0.443**	0.364**	-0.442**	-0.693**	-0.772**	-0.231
	**Sig. (2-tailed)**	0.001	0.073	0.005		0.671	0.000	0.004	0.000	0.000	0.000	0.076
	**N**	60	60	60	60	60	60	60	60	60	60	60
**Density**	**Pearson Coefficient**	-0.009	0.014	0.000	0.056	1	-0.029	0.158	-0.050	-0.104	0.035	0.017
	**Sig. (2-tailed)**	0.945	0.916	0.998	0.671		0.825	0.228	0.703	0.428	0.788	0.897
	**N**	60	60	60	60	60	60	60	60	60	60	60
**PMI*x***	**Pearson Coefficient**	0.819**	-0.280*	-0.828**	0.443**	-0.029	1	0.721**	-0.953**	-0.042	-0.260*	-0.027
	**Sig. (2-tailed)**	0.000	0.030	0.000	0.000	0.825		0.000	0.000	0.751	0.045	0.841
	**N**	60	60	60	60	60	60	60	60	60	60	60
**PMI*y***	**Pearson Coefficient**	0.740**	-0.613**	-0.453**	0.364**	0.158	0.721**	1	-0.897**	0.068	-0.105	-0.131
	**Sig. (2-tailed)**	0.000	0.000	0.000	0.004	0.228	0.000		0.000	0.605	0.426	0.317
	**N**	60	60	60	60	60	60	60	60	60	60	60
**PMI*z***	**Pearson Coefficient**	-0.846**	0.447**	0.726**	-0.442**	-0.050	-0.953**	-0.897**	1	-0.005	0.221	0.080
	**Sig. (2-tailed)**	0.000	0.000	0.000	0.000	0.703	0.000	0.000		0.970	0.090	0.543
	**N**	60	60	60	60	60	60	60	60	60	60	60
**Height**	**Spearman Coefficient**	0.024	0.052	-0.013	-0.693**	-0.104	-0.042	0.068	-0.005	1	0.788**	0.259*
	**Sig. (2-tailed)**	0.855	0.693	0.922	0.000	0.428	0.751	0.605	0.970		0.000	0.045
	**N**	60	60	60	60	60	60	60	60	60	60	60
**Weight**	**Spearman Coefficient**	-0.162	0.090	0.173	-0.772**	0.035	-0.260*	-0.105	0.221	0.788**	1	0.198
	**Sig. (2-tailed)**	0.215	0.492	0.187	0.000	0.788	0.045	0.426	0.090	0.000		0.130
	**N**	60	60	60	60	60	60	60	60	60	60	60
**Age**	**Spearman Coefficient**	-0.104	0.276*	-0.043	-0.231	0.017	-0.027	-0.131	0.080	0.259*	0.198	1
	**Sig. (2-tailed)**	0.429	0.033	0.743	0.076	0.897	0.841	0.317	0.543	0.045	0.130	
	**N**	60	60	60	60	60	60	60	60	60	60	60

Table S10 Correlation results between variables of the 5th metatarsal of the left side

		Length	Width	Height	S: V	Density	PMI*x*	PMI*y*	PMIz	Height	Weight	Age
**Length**	**Pearson Coefficient**	1	-0.804**	-0.773**	0.483**	-0.238	0.766**	0.802**	-0.837**	-0.113	-0.296*	-0.331**
	**Sig. (2-tailed)**		0.000	0.000	0.000	0.067	0.000	0.000	0.000	0.390	0.022	0.010
	**N**	60	60	60	60	60	60	60	60	60	60	60
**Width**	**Pearson Coefficient**	-0.804**	1	0.245	-0.319*	0.118	-0.575**	-0.849**	0.791**	-0.067	0.105	0.272*
	**Sig. (2-tailed)**	0.000		0.059	0.013	0.371	0.000	0.000	0.000	0.609	0.426	0.036
	**N**	60	60	60	60	60	60	60	60	60	60	60
**Height**	**Pearson Coefficient**	-0.773**	0.245	1	-0.447**	0.263*	-0.636**	-0.404**	0.522**	0.243	0.353**	0.249
	**Sig. (2-tailed)**	0.000	0.059		0.000	0.043	0.000	0.001	0.000	0.062	0.006	0.055
	**N**	60	60	60	60	60	60	60	60	60	60	60
**S: V**	**Pearson Coefficient**	0.483**	-0.319*	-0.447**	1	0.042	0.351**	0.440**	-0.431**	-0.674**	-0.797**	-0.327*
	**Sig. (2-tailed)**	0.000	0.013	0.000		0.749	0.006	0.000	0.001	0.000	0.000	0.011
	**N**	60	60	60	60	60	60	60	60	60	60	60
**Density**	**Pearson Coefficient**	-0.238	0.118	0.263*	0.042	1	0.044	-0.038	0.008	-0.116	0.013	-0.032
	**Sig. (2-tailed)**	0.067	0.371	0.043	0.749		0.789	0.771	0.954	0.379	0.921	0.807
	**N**	60	60	60	60	60	60	60	60	60	60	60
**PMI*x***	**Pearson Coefficient**	0.766**	-0.575**	-0.636**	0.351**	0.044	1	0.760**	-0.904**	0.073	-0.136	-0.105
	**Sig. (2-tailed)**	0.000	0.000	0.000	0.006	0.789		0.000	0.000	0.577	0.299	0.426
	**N**	60	60	60	60	60	60	60	60	60	60	60
**PMI*y***	**Pearson Coefficient**	0.802**	-0.849**	-0.404**	0.440**	-0.038	0.760**	1	-0.965**	0.002	-0.179	-0.225
	**Sig. (2-tailed)**	0.000	0.000	0.001	0.000	0.771	0.000		0.000	0.991	0.172	0.084
	**N**	60	60	60	60	60	60	60	60	60	60	60
**PMIz**	**Pearson Coefficient**	-0.837**	0.791**	0.522**	-0.431**	0.008	-0.904**	-0.965**	1	-0.076	0.158	0.174
	**Sig. (2-tailed)**	0.000	0.000	0.000	0.001	0.954	0.000	0.000		0.563	0.228	0.184
	**N**	60	60	60	60	60	60	60	60	60	60	60
**Height**	**Spearman Coefficient**	-0.113	-0.067	0.243	-0.674**	-0.116	0.073	0.002	-0.076	1	0.788**	0.259*
	**Sig. (2-tailed)**	0.390	0.609	0.062	0.000	0.379	0.577	0.991	0.563		0.000	0.045
	**N**	60	60	60	60	60	60	60	60	60	60	60
**Weight**	**Spearman Coefficient**	-0.296*	0.105	0.353**	-0.797**	0.013	-0.136	-0.179	0.158	0.788**	1	0.198
	**Sig. (2-tailed)**	0.022	0.426	0.006	0.000	0.921	0.299	0.172	0.228	0.000		0.130
	**N**	60	60	60	60	60	60	60	60	60	60	60
**Age**	**Spearman Coefficient**	-0.331**	0.272*	0.249	-0.327*	-0.032	-0.105	-0.225	0.174	0.259*	0.198	1
	**Sig. (2-tailed)**	0.010	0.036	0.055	0.011	0.807	0.426	0.084	0.184	0.045	0.130	
	**N**	60	60	60	60	60	60	60	60	60	60	60

Table S11 Correlation results between variables of the 1st metatarsal of the right side

		Length	Width	Height	S: V	Density	PMI*x*	PMI*y*	PMIz	Height	Weight	Age
**Length**	**Pearson Coefficient**	1	-0.823**	-0.875**	0.320*	-0.106	0.802**	0.646**	-0.866**	0.033	-0.294*	-0.011
	**Sig. (2-tailed)**		0.000	0.000	0.013	0.419	0.000	0.000	0.000	0.800	0.023	0.931
	**N**	60	60	60	60	60	60	60	60	60	60	60
**Width**	**Pearson Coefficient**	-0.823**	1	0.444**	-0.291*	0.130	-0.602**	-0.713**	0.782**	0.121	0.363**	0.041
	**Sig. (2-tailed)**	0.000		0.000	0.024	0.324	0.000	0.000	0.000	0.355	0.004	0.754
	**N**	60	60	60	60	60	60	60	60	60	60	60
**Height**	**Pearson Coefficient**	-0.875**	0.444**	1	-0.257*	0.057	-0.751**	-0.409**	0.698**	-0.114	0.206	-0.066
	**Sig. (2-tailed)**	0.000	0.000		0.048	0.665	0.000	0.001	0.000	0.385	0.114	0.614
	**N**	60	60	60	60	60	60	60	60	60	60	60
**S: V**	**Pearson Coefficient**	0.320*	-0.291*	-0.257*	1	0.130	0.302*	0.396**	-0.414**	-0.768**	-0.777**	-0.261*
	**Sig. (2-tailed)**	0.013	0.024	0.048		0.320	0.019	0.002	0.001	0.000	0.000	0.044
	**N**	60	60	60	60	60	60	60	60	60	60	60
**Density**	**Pearson Coefficient**	-0.106	0.130	0.057	0.130	1	-0.104	-0.020	0.076	-0.072	-0.007	-0.070
	**Sig. (2-tailed)**	0.419	0.324	0.665	0.320		0.429	0.878	0.565	0.583	0.960	0.596
	**N**	60	60	60	60	60	60	60	60	60	60	60
**PMI*x***	**Pearson Coefficient**	0.802**	-0.602**	-0.751**	0.302*	-0.104	1	0.406**	-0.849**	0.123	-0.129	-0.005
	**Sig. (2-tailed)**	0.000	0.000	0.000	0.019	0.429		0.001	0.000	0.351	0.327	0.970
	**N**	60	60	60	60	60	60	60	60	60	60	60
**PMI*y***	**Pearson Coefficient**	0.646**	-0.713**	-0.409**	0.396**	-0.020	0.406**	1	-0.827**	-0.154	-0.345**	-0.067
	**Sig. (2-tailed)**	0.000	0.000	0.001	0.002	0.878	0.001		0.000	0.239	0.007	0.610
	**N**	60	60	60	60	60	60	60	60	60	60	60
**PMIz**	**Pearson Coefficient**	-0.866**	0.782**	0.698**	-0.414**	0.076	-0.849**	-0.827**	1	0.009	0.288*	0.067
	**Sig. (2-tailed)**	0.000	0.000	0.000	0.001	0.565	0.000	0.000		0.947	0.026	0.612
	**N**	60	60	60	60	60	60	60	60	60	60	60
**Height**	**Spearman Coefficient**	0.033	0.121	-0.114	-0.768**	-0.072	0.123	-0.154	0.009	1	0.788**	0.259*
	**Sig. (2-tailed)**	0.800	0.355	0.385	0.000	0.583	0.351	0.239	0.947		0.000	0.045
	**N**	60	60	60	60	60	60	60	60	60	60	60
**Weight**	**Spearman Coefficient**	-0.294*	0.363**	0.206	-0.777**	-0.007	-0.129	-0.345**	0.288*	0.788**	1	0.198
	**Sig. (2-tailed)**	0.023	0.004	0.114	0.000	0.960	0.327	0.007	0.026	0.000		0.130
	**N**	60	60	60	60	60	60	60	60	60	60	60
**Age**	**Spearman Coefficient**	-0.011	0.041	-0.066	-0.261*	-0.070	-0.005	-0.067	0.067	0.259*	0.198	1
	**Sig. (2-tailed)**	0.931	0.754	0.614	0.044	0.596	0.970	0.610	0.612	0.045	0.130	
	**N**	60	60	60	60	60	60	60	60	60	60	60

Table S12 Correlation results between variables of the 2nd metatarsal of the right side

		Length	Width	Height	S: V	Density	PMI*x*	PMI*y*	PMI*z*	Height	Weight	Age
**Length**	**Pearson Coefficient**	1	-0.766**	-0.835**	0.218	-0.140	0.844**	0.796**	-0.873**	0.144	-0.100	-0.113
	**Sig. (2-tailed)**		0.000	0.000	0.094	0.286	0.000	0.000	0.000	0.273	0.449	0.390
	**N**	60	60	60	60	60	60	60	60	60	60	60
**Width**	**Pearson Coefficient**	-0.766**	1	0.286*	-0.213	0.062	-0.502**	-0.735**	0.625**	-0.051	0.036	0.187
	**Sig. (2-tailed)**	0.000		0.026	0.102	0.638	0.000	0.000	0.000	0.696	0.787	0.153
	**N**	60	60	60	60	60	60	60	60	60	60	60
**Height**	**Pearson Coefficient**	-0.835**	0.286*	1	-0.143	0.156	-0.829**	-0.558**	0.766**	-0.146	0.088	0.011
	**Sig. (2-tailed)**	0.000	0.026		0.275	0.235	0.000	0.000	0.000	0.266	0.503	0.933
	**N**	60	60	60	60	60	60	60	60	60	60	60
**S: V**	**Pearson Coefficient**	0.218	-0.213	-0.143	1	0.109	0.423**	0.501**	-0.479**	-0.775**	-0.792**	-0.231
	**Sig. (2-tailed)**	0.094	0.102	0.275		0.409	0.001	0.000	0.000	0.000	0.000	0.076
	**N**	60	60	60	60	60	60	60	60	60	60	60
**Density**	**Pearson Coefficient**	-0.140	0.062	0.156	0.109	1	0.038	0.126	-0.076	-0.096	-0.124	-0.142
	**Sig. (2-tailed)**	0.286	0.638	0.235	0.409		0.776	0.339	0.566	0.467	0.344	0.278
	**N**	60	60	60	60	60	60	60	60	60	60	60
**PMI*x***	**Pearson Coefficient**	0.844**	-0.502**	-0.829**	0.423**	0.038	1	0.778**	-0.967**	-0.096	-0.361**	-0.081
	**Sig. (2-tailed)**	0.000	0.000	0.000	0.001	0.776		0.000	0.000	0.465	0.005	0.537
	**N**	60	60	60	60	60	60	60	60	60	60	60
**PMI*y***	**Pearson Coefficient**	0.796**	-0.735**	-0.558**	0.501**	0.126	0.778**	1	-0.913**	-0.128	-0.349**	-0.171
	**Sig. (2-tailed)**	0.000	0.000	0.000	0.000	0.339	0.000		0.000	0.329	0.006	0.192
	**N**	60	60	60	60	60	60	60	60	60	60	60
**PMI*z***	**Pearson Coefficient**	-0.873**	0.625**	0.766**	-0.479**	-0.076	-0.967**	-0.913**	1	0.108	0.375**	0.112
	**Sig. (2-tailed)**	0.000	0.000	0.000	0.000	0.566	0.000	0.000		0.411	0.003	0.394
	**N**	60	60	60	60	60	60	60	60	60	60	60
**Height**	**Spearman Coefficient**	0.144	-0.051	-0.146	-0.775**	-0.096	-0.096	-0.128	0.108	1	0.788**	0.259*
	**Sig. (2-tailed)**	0.273	0.696	0.266	0.000	0.467	0.465	0.329	0.411		0.000	0.045
	**N**	60	60	60	60	60	60	60	60	60	60	60
**Weight**	**Spearman Coefficient**	-0.100	0.036	0.088	-0.792**	-0.124	-0.361**	-0.349**	0.375**	0.788**	1	0.198
	**Sig. (2-tailed)**	0.449	0.787	0.503	0.000	0.344	0.005	0.006	0.003	0.000		0.130
	**N**	60	60	60	60	60	60	60	60	60	60	60
**Age**	**Spearman Coefficient**	-0.113	0.187	0.011	-0.231	-0.142	-0.081	-0.171	0.112	0.259*	0.198	1
	**Sig. (2-tailed)**	0.390	0.153	0.933	0.076	0.278	0.537	0.192	0.394	0.045	0.130	
	**N**	60	60	60	60	60	60	60	60	60	60	60

Table S13 Correlation results between variables of the 3rd metatarsal of the right side

		Length	Width	Height	S: V	Density	PMI*x*	PMI*y*	PMIz	Height	Weight	Age
**Length**	**Pearson Coefficient**	1	-0.727**	-0.839**	0.321*	-0.150	0.837**	0.792**	-0.865**	0.102	-0.171	-0.004
	**Sig. (2-tailed)**		0.000	0.000	0.012	0.252	0.000	0.000	0.000	0.438	0.191	0.975
	**N**	60	60	60	60	60	60	60	60	60	60	60
**Width**	**Pearson Coefficient**	-0.727**	1	0.236	-0.317*	0.130	-0.453**	-0.718**	0.581**	-0.043	0.021	0.149
	**Sig. (2-tailed)**	0.000		0.069	0.014	0.323	0.000	0.000	0.000	0.744	0.872	0.255
	**N**	60	60	60	60	60	60	60	60	60	60	60
**Height**	**Pearson Coefficient**	-0.839**	0.236	1	-0.203	0.109	-0.825**	-0.553**	0.764**	-0.135	0.175	-0.118
	**Sig. (2-tailed)**	0.000	0.069		0.119	0.405	0.000	0.000	0.000	0.305	0.182	0.371
	**N**	60	60	60	60	60	60	60	60	60	60	60
**S: V**	**Pearson Coefficient**	0.321*	-0.317*	-0.203	1	0.063	0.459**	0.486**	-0.495**	-0.681**	-0.808**	-0.271*
	**Sig. (2-tailed)**	0.012	0.014	0.119		0.633	0.000	0.000	0.000	0.000	0.000	0.036
	**N**	60	60	60	60	60	60	60	60	60	60	60
**Density**	**Pearson Coefficient**	-0.150	0.130	0.109	0.063	1	0.001	0.001	0.001	-0.120	-0.039	0.055
	**Sig. (2-tailed)**	0.252	0.323	0.405	0.633		0.996	0.991	0.994	0.361	0.769	0.676
	**N**	60	60	60	60	60	60	60	60	60	60	60
**PMI*x***	**Pearson Coefficient**	0.837**	-0.453**	-0.825**	0.459**	0.001	1	0.784**	-0.970**	-0.002	-0.352**	-0.023
	**Sig. (2-tailed)**	0.000	0.000	0.000	0.000	0.996		0.000	0.000	0.989	0.006	0.862
	**N**	60	60	60	60	60	60	60	60	60	60	60
**PMI*y***	**Pearson Coefficient**	0.792**	-0.718**	-0.553**	0.486**	0.001	0.784**	1	-0.911**	-0.041	-0.249	-0.092
	**Sig. (2-tailed)**	0.000	0.000	0.000	0.000	0.991	0.000		0.000	0.757	0.055	0.482
	**N**	60	60	60	60	60	60	60	60	60	60	60
**PMIz**	**Pearson Coefficient**	-0.865**	0.581**	0.764**	-0.495**	0.001	-0.970**	-0.911**	1	0.015	0.343**	0.066
	**Sig. (2-tailed)**	0.000	0.000	0.000	0.000	0.994	0.000	0.000		0.907	0.007	0.618
	**N**	60	60	60	60	60	60	60	60	60	60	60
**Height**	**Spearman Coefficient**	0.102	-0.043	-0.135	-0.681**	-0.120	-0.002	-0.041	0.015	1	0.788**	0.259*
	**Sig. (2-tailed)**	0.438	0.744	0.305	0.000	0.361	0.989	0.757	0.907		0.000	0.045
	**N**	60	60	60	60	60	60	60	60	60	60	60
**Weight**	**Spearman Coefficient**	-0.171	0.021	0.175	-0.808**	-0.039	-0.352**	-0.249	0.343**	0.788**	1	0.198
	**Sig. (2-tailed)**	0.191	0.872	0.182	0.000	0.769	0.006	0.055	0.007	0.000		0.130
	**N**	60	60	60	60	60	60	60	60	60	60	60
**Age**	**Spearman Coefficient**	-0.004	0.149	-0.118	-0.271*	0.055	-0.023	-0.092	0.066	0.259*	0.198	1
	**Sig. (2-tailed)**	0.975	0.255	0.371	0.036	0.676	0.862	0.482	0.618	0.045	0.130	
	**N**	60	60	60	60	60	60	60	60	60	60	60

Table S14 Correlation results between variables of the 4th metatarsal of the right side

		Length	Width	Height	S: V	Density	PMI*x*	PMI*y*	PMIz	Height	Weight	Age
**Length**	**Pearson Coefficient**	1	-0.718**	-0.857**	0.404**	-0.003	0.834**	0.682**	-0.888**	0.027	-0.151	-0.093
	**Sig. (2-tailed)**		0.000	0.000	0.001	0.984	0.000	0.000	0.000	0.839	0.248	0.478
	**N**	60	60	60	60	60	60	60	60	60	60	60
**Width**	**Pearson Coefficient**	-0.718**	1	0.256*	-0.370**	-0.073	-0.404**	-0.770**	0.631**	0.045	0.114	0.088
	**Sig. (2-tailed)**	0.000		0.048	0.004	0.580	0.001	0.000	0.000	0.732	0.387	0.505
	**N**	60	60	60	60	60	60	60	60	60	60	60
**Height**	**Pearson Coefficient**	-0.857**	0.256*	1	-0.287*	0.058	-0.859**	-0.377**	0.765**	-0.031	0.157	0.024
	**Sig. (2-tailed)**	0.000	0.048		0.026	0.662	0.000	0.003	0.000	0.813	0.231	0.855
	**N**	60	60	60	60	60	60	60	60	60	60	60
**S: V**	**Pearson Coefficient**	0.404**	-0.370**	-0.287*	1	0.138	0.438**	0.393**	-0.483**	-0.709**	-0.765**	-0.260*
	**Sig. (2-tailed)**	0.001	0.004	0.026		0.292	0.000	0.002	0.000	0.000	0.000	0.045
	**N**	60	60	60	60	60	60	60	60	60	60	60
**Density**	**Pearson Coefficient**	-0.003	-0.073	0.058	0.138	1	-0.001	0.303*	-0.124	-0.033	0.125	0.054
	**Sig. (2-tailed)**	0.984	0.580	0.662	0.292		0.992	0.020	0.349	0.802	0.341	0.679
	**N**	60	60	60	60	60	60	60	60	60	60	60
**PMI*x***	**Pearson Coefficient**	0.834**	-0.404**	-0.859**	0.438**	-0.001	1	0.498**	-0.918**	-0.054	-0.288*	-0.133
	**Sig. (2-tailed)**	0.000	0.001	0.000	0.000	0.992		0.000	0.000	0.683	0.026	0.312
	**N**	60	60	60	60	60	60	60	60	60	60	60
**PMI*y***	**Pearson Coefficient**	0.682**	-0.770**	-0.377**	0.393**	0.303*	0.498**	1	-0.801**	0.069	-0.086	-0.094
	**Sig. (2-tailed)**	0.000	0.000	0.003	0.002	0.020	0.000		0.000	0.599	0.513	0.477
	**N**	60	60	60	60	60	60	60	60	60	60	60
**PMIz**	**Pearson Coefficient**	-0.888**	0.631**	0.765**	-0.483**	-0.124	-0.918**	-0.801**	1	-0.001	0.227	0.098
	**Sig. (2-tailed)**	0.000	0.000	0.000	0.000	0.349	0.000	0.000		0.997	0.081	0.457
	**N**	60	60	60	60	60	60	60	60	60	60	60
**Height**	**Spearman Coefficient**	0.027	0.045	-0.031	-0.709**	-0.033	-0.054	0.069	-0.001	1	0.788**	0.259*
	**Sig. (2-tailed)**	0.839	0.732	0.813	0.000	0.802	0.683	0.599	0.997		0.000	0.045
	**N**	60	60	60	60	60	60	60	60	60	60	60
**Weight**	**Spearman Coefficient**	-0.151	0.114	0.157	-0.765**	0.125	-0.288*	-0.086	0.227	0.788**	1	0.198
	**Sig. (2-tailed)**	0.248	0.387	0.231	0.000	0.341	0.026	0.513	0.081	0.000		0.130
	**N**	60	60	60	60	60	60	60	60	60	60	60
**Age**	**Spearman Coefficient**	-0.093	0.088	0.024	-0.260*	0.054	-0.133	-0.094	0.098	0.259*	0.198	1
	**Sig. (2-tailed)**	0.478	0.505	0.855	0.045	0.679	0.312	0.477	0.457	0.045	0.130	
	**N**	60	60	60	60	60	60	60	60	60	60	60

Table S15 Correlation results between variables of the 5th metatarsal of the right side

		Length	Width	Height	S: V	Density	PMI*x*	PMI*y*	PMIz	Height	Weight	Age
**Length**	**Pearson Coefficient**	1	-0.782**	-0.677**	0.377**	-0.070	0.814**	0.790**	-0.844**	-0.011	-0.294*	-0.198
	**Sig. (2-tailed)**		0.000	0.000	0.003	0.594	0.000	0.000	0.000	0.931	0.023	0.129
	**N**	60	60	60	60	60	60	60	60	60	60	60
**Width**	**Pearson Coefficient**	-0.782**	1	0.071	-0.275*	-0.094	-0.609**	-0.853**	0.804**	-0.003	0.152	0.129
	**Sig. (2-tailed)**	0.000		0.591	0.033	0.474	0.000	0.000	0.000	0.981	0.245	0.326
	**N**	60	60	60	60	60	60	60	60	60	60	60
**Height**	**Pearson Coefficient**	-0.677**	0.071	1	-0.279*	0.224	-0.583**	-0.257*	0.401**	0.011	0.240	0.182
	**Sig. (2-tailed)**	0.000	0.591		0.031	0.086	0.000	0.048	0.002	0.932	0.065	0.165
	**N**	60	60	60	60	60	60	60	60	60	60	60
**S: V**	**Pearson Coefficient**	0.377**	-0.275*	-0.279*	1	0.060	0.315*	0.381**	-0.376**	-0.710**	-0.809**	-0.278*
	**Sig. (2-tailed)**	0.003	0.033	0.031		0.649	0.014	0.003	0.003	0.000	0.000	0.031
	**N**	60	60	60	60	60	60	60	60	60	60	60
**Density**	**Pearson Coefficient**	-0.070	-0.094	0.224	0.060	1	0.096	0.204	-0.173	0.003	0.100	0.122
	**Sig. (2-tailed)**	0.594	0.474	0.086	0.649		0.464	0.117	0.186	0.981	0.448	0.353
	**N**	60	60	60	60	60	60	60	60	60	60	60
**PMI*x***	**Pearson Coefficient**	0.814**	-0.609**	-0.583**	0.315*	0.096	1	0.779**	-0.911**	0.153	-0.120	-0.059
	**Sig. (2-tailed)**	0.000	0.000	0.000	0.014	0.464		0.000	0.000	0.244	0.360	0.652
	**N**	60	60	60	60	60	60	60	60	60	60	60
**PMI*y***	**Pearson Coefficient**	0.790**	-0.853**	-0.257*	0.381**	0.204	0.779**	1	-0.968**	0.026	-0.173	-0.068
	**Sig. (2-tailed)**	0.000	0.000	0.048	0.003	0.117	0.000		0.000	0.845	0.185	0.606
	**N**	60	60	60	60	60	60	60	60	60	60	60
**PMIz**	**Pearson Coefficient**	-0.844**	0.804**	0.401**	-0.376**	-0.173	-0.911**	-0.968**	1	-0.095	0.163	0.066
	**Sig. (2-tailed)**	0.000	0.000	0.002	0.003	0.186	0.000	0.000		0.470	0.213	0.617
	**N**	60	60	60	60	60	60	60	60	60	60	60
**Height**	**Spearman Coefficient**	-0.011	-0.003	0.011	-0.710**	0.003	0.153	0.026	-0.095	1	0.788**	0.259*
	**Sig. (2-tailed)**	0.931	0.981	0.932	0.000	0.981	0.244	0.845	0.470		0.000	0.045
	**N**	60	60	60	60	60	60	60	60	60	60	60
**Weight**	**Spearman Coefficient**	-0.294*	0.152	0.240	-0.809**	0.100	-0.120	-0.173	0.163	0.788**	1	0.198
	**Sig. (2-tailed)**	0.023	0.245	0.065	0.000	0.448	0.360	0.185	0.213	0.000		0.130
	**N**	60	60	60	60	60	60	60	60	60	60	60
**Age**	**Spearman Coefficient**	-0.198	0.129	0.182	-0.278*	0.122	-0.059	-0.068	0.066	0.259*	0.198	1
	**Sig. (2-tailed)**	0.129	0.326	0.165	0.031	0.353	0.652	0.606	0.617	0.045	0.130	
	**N**	60	60	60	60	60	60	60	60	60	60	60

Table S16 Homogeneity of variance-covariance matrices results

Sides	Box's test	Metatarsals				
		1st	2nd	3rd	4th	5th
**Left**	**Box's M**	2.845	4.764	0.764	3.253	7.501
	**F**	0.447	1.529	0.245	3.198	2.407
	**sig**	0.848	0.205	0.865	0.074	0.065
**Right**	**Box's M**	10.088	14.540	2.663	12.795	7.860
	**F**	1.585	4.666	0.855	2.012	2.522
	**sig**	0.147	0.003	0.464	0.060	0.056

Table S17 Mardia's multivariate skewness and kurtosis results

Sides	Metatarsals	Skewness			skewness		
		**b**	**z**	***sig***	**b**	**z**	***sig***
**Left**	**1st**	3.637	35.759	0.433	34.460	-0.248	0.804
	**2nd**	6.181	61.811	0.003**	37.602	1.205	0.228
	**3rd**	5.287	52.869	0.027*	37.970	1.375	0.169
	**4th**	5.673	56.732	0.012*	35.863	0.340	0.689
	**5th**	5.684	56.838	0.011*	39.048	1.874	0.061
**Right**	**1st**	3.836	37.721	0.346	30.432	-2.097	0.036*
	**2nd**	4.644	46.441	0.094	36.712	0.792	0.428
	**3rd**	4.079	40.794	0.231	34.754	-0.114	0.909
	**4th**	4.139	41.395	0.212	34.330	-0.310	0.757
	**5th**	6.485	64.849	0.002**	40.315	2.460	0.014*

* Significance level: $p < 0.05$; ** Significance level: $p < 0.01$;

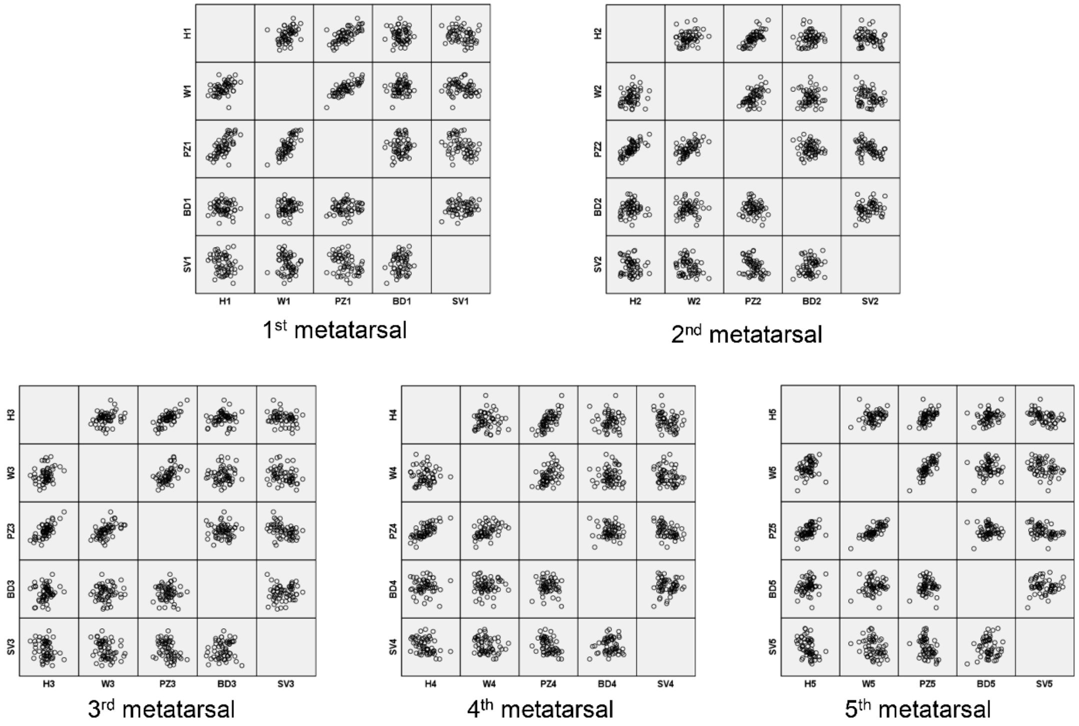

Figure S5-A Linearity of five variables of the 1st - 5th metatarsal bones of the left side

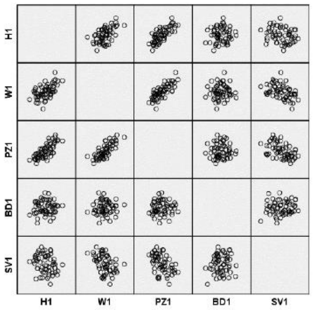

1st metatarsal

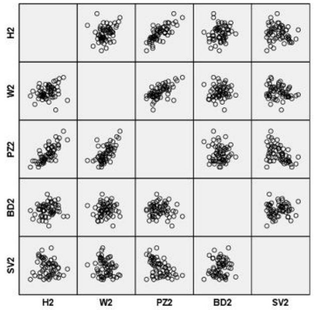

2nd metatarsal

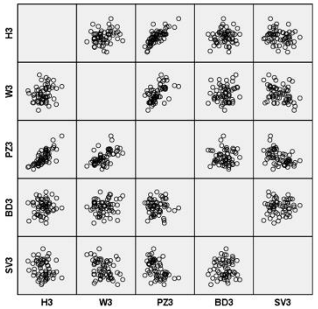

3rd metatarsal

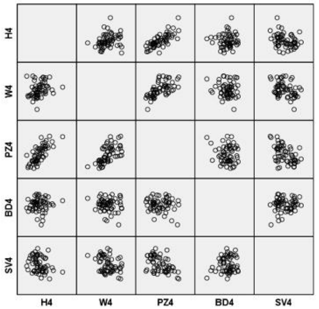

4th metatarsal

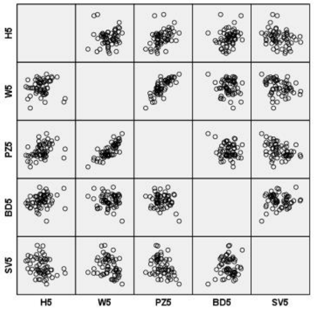

5th metatarsal

Figure S5-B Linearity of five variables of the 1st - 5th metatarsal bones of the right side
